# Indirect social contact interventions to reduce mental health-related stigma in low- and middle-income countries: systematic review

**DOI:** 10.1017/S2045796022000622

**Published:** 2022-11-09

**Authors:** A. Makhmud, G. Thornicroft, P. C. Gronholm

**Affiliations:** Centre for Global Mental Health and Centre for Implementation Science, Institute of Psychiatry, Psychology and Neuroscience, King's College London, London, UK

**Keywords:** Discrimination, mental health, mental illness stigma, systematic reviews

## Abstract

**Aims:**

Mental health-related stigma and discrimination are a complex and widespread issue with negative effects on numerous aspects of life of people with lived experience of mental health conditions. Research shows that social contact is the best evidence-based intervention to reduce stigma. Within the context of a rapid development of remote technology, and COVID-19-related restrictions for face-to-face contact, the aim of this paper is to categorise, compare and define indirect social contact (ISC) interventions to reduce stigma and discrimination in mental health in low- and middle-income countries (LMICs).

**Methods:**

MEDLINE, Global Health, EMBASE, PsychINFO, Cochrane Central Register of Control Trials (CENTRAL), Cumulative Index to Nursing and Allied Health Literature (CINAHL) were searched using a strategy including terms related to ‘stigma and discrimination’, ‘intervention’, ‘indirect social contact’, ‘mental health’ and ‘low- and middle-income countries’. Relevant information on ISC interventions was extracted from the included articles, and a quality assessment was conducted. Emerging themes were coded using a thematic synthesis method, and a narrative synthesis was undertaken to present the results.

**Results:**

Nine studies were included in the review overall. One study was ineffective; this was not considered for the categorisation of interventions, and it was considered separately for the comparison of interventions. Of the eight effective studies included in synthesis, interventions were categorised by content, combination of stigma-reducing strategies, medium of delivery, delivery agents, target condition and population, as well as by active or passive interaction and follow-up. Most of the interventions used education and ISC. Recovery and personal experience were important content components as all studies included either one or both. Cultural adaptation and local relevance were also important considerations.

**Conclusions:**

ISC interventions were effective in overall terms for both the general public and healthcare providers, including medical students. A new definition of ISC interventions in LMICs is proposed. More research and better reporting of intervention details are needed to explore the effectiveness of ISC strategies in LMICs, especially in regions where little relevant research has been conducted.

## Introduction

Stigma and discrimination are a complex and multifaceted phenomenon conceptualised in various ways across disciplines and literature. Conceptualisation of stigma includes problems related to knowledge (ignorance), attitude (prejudice) and behaviour (discrimination) (Thornicroft *et al*., 2007). This definition is evident also in how stigma is assessed using measures capturing these aspects of knowledge (e.g. Mental Health Knowledge Schedule (Evans-Lacko *et al*., 2010)), attitudes (e.g. Community Attitudes to Mental Illness scale (Wolff *et al*., 1996)) and behaviour (e.g. Reported and Intended Behaviour Scale (Evans-Lacko *et al*., 2011)).

Stigma and discrimination may vary between cultures but are prevalent in all regions of the world (Thornicroft *et al*., 2009; Koschorke *et al*., 2017; Winkler *et al*., 2017; Aliev *et al*., 2021). Stigma is a major part of frequently experienced personal distress, systematic disadvantages, economic loss and social exclusion linked to mental illness globally. The negative impacts of stigma can have widespread effects on the personal (Corrigan *et al*., 2006; Thornicroft *et al*., 2009), social (Yang *et al*., 2007; Gonsalves *et al*., 2019), economic (Sharac *et al*., 2010) and other (Clement *et al*., 2011, 2015) aspects of lives of people with mental health needs. Also, stigma and discrimination have important implications in policy with low investment, and political commitment towards mental healthcare programmes (Saraceno *et al*., 2007) which reflect structural stigma (Pescosolido and Martin, 2015).

Strategies to reduce mental health-related stigma can use education, protest or social contact approaches (Corrigan *et al*., 2001). Educational approaches targeting knowledge and beliefs about mental health-related problems have been shown to be effective and widely used (Mehta *et al*., 2015; Thornicroft *et al*., 2016; Rao *et al*., 2019). Protest strategies challenge negative representation and images of mental illness and people with mental health needs (Corrigan *et al*., 2001). However, evidence on their effectiveness is limited (Corrigan *et al*., 2012). Social contact involves contact between the stigmatised group and those displaying stigmatising attitudes, knowledge or behaviour (London and Evans-Lacko, 2010), and is the most effective type of intervention to reduce mental health-related stigma and discrimination (Thornicroft *et al*., 2016).

Social contact interventions targeting mental health-related stigma take various forms and cover a range of intervention types. More recently, *indirect social contact (ISC)* interventions that do not entail in-person face-to-face contact have been developed and evaluated. ISC interventions have been broadly divided into those occurring through: (i) another person (e.g. someone who knows a person with mental health needs), (ii) media (e.g. the Internet) or (iii) imagined contact, or having passive or active interaction with ISC media (e.g. discussing videos or vignettes) (Paolini *et al*., 2021).

Yet there is a limited understanding of what defines ISC interventions or how various types of ISC differ. Some reviews have explored the effects of intergroup social contact (Maunder and White, 2019) or effects of certain types of ISC (Ando *et al*., 2011; Janoušková *et al*., 2017), but no systematic reviews defining and comparing ISC in low- and middle-income countries (LMICs) have been published. A focus on LMICs is important because of the broader mental health research and evidence gap on contact-based intervention in such contexts, and because the large majority of the world's population live in LMICs (Thornicroft *et al*., 2016).

The COVID-19 pandemic has led to an increase in risk factors for mental health conditions and exacerbated barriers to support for people with pre-existing mental health needs (Moreno *et al*., 2020). Effects of the pandemic on mental health are more prominent in LMICs given other local endemics, stigma and pre-existing difficulties in mental healthcare (De Sousa *et al*., 2020). ISC can be useful to target mental health-related stigma under current circumstances where face-to-face contact is restricted and the mental health burden is rising (Vigo *et al*., 2020; Naslund and Deng, 2021).

The aims of this systematic review are to address the gap in research on ISC in LMICs by categorising, comparing and defining ISC interventions to reduce mental health-related stigma in LMICs.

## Methods

### Inclusion and exclusion criteria

The protocol for the systematic review was registered on PROSPERO, ID CRD42021248559. This review included studies with interventions containing an element of ISC aimed to reduce any type of mental health-related stigma or discrimination against people with mental health needs. Studies that focused on other stigmatised conditions such as HIV, substance use disorders and neurological conditions were excluded. ISC of any kind – including for example videos, presentations, personal narratives, photo-voice and theatrical performances – were eligible for inclusion. Comparators such as a non-exposed control group or a control group exposed to another type of stigma-reducing intervention were included as long as the effect of ISC specifically or its effect alongside one other stigma-reducing intervention could be analysed. Studies that did not have a comparator or control group were included as long as outcome measures were taken pre- and post-intervention. In this review, studies were considered to assess stigma if they explicitly stated they assessed stigma, or also if they captured stigma via the constructs of knowledge, attitudes and/or behaviour. Studies of all experimental designs were eligible for this review as long as at least one mental health-related stigma measure was collected pre and post intervention. Studies eligible for this review must have been conducted in a country classified as LMIC by the World Bank classification of gross national income (2019). Studies of any duration, size or follow-up were included. No restrictions were applied on target populations and publishing date. Searches were restricted to English language, and to human subjects.

### Search strategy

The search strategy development was guided by other systematic reviews on stigma (Mehta *et al*., 2015; Morgan *et al*., 2018; Clay *et al*., 2020; Heim *et al*., 2020). Five categories of terms (‘stigma’, ‘intervention’, ‘indirect social contact’, ‘mental health’ and ‘low- and middle-income countries’) were expanded with related subject headings and key words, connected with ‘OR’ within categories and ‘AND’ between categories. The full search strategy for databases used is provided within online Supplementary materials.

Records from MEDLINE, Global Health, EMBASE, PsychINFO, The Cochrane Central Register of Controlled Trials (CENTRAL), Cumulative Index to Nursing and Allied Health Literature (CINAHL) were retrieved on 29 June 2021. In our review protocol we indicated that we could also conduct the search in Scopus; however, due to issues with feasibility and system errors at the time of the searches, Scopus was not used.

In addition to the database search, we performed backward and forward citation checking of included papers and checked reference lists of related systematic reviews (can be accessed in online Supplementary materials). Authors of included studies and other content experts were contacted for paper recommendations.

### Study selection

All titles and abstracts were screened for potential relevance by the lead author, and 17% titles and abstracts were independently screened by the second reviewer to establish consistency. Reviewers resolved any disagreements through discussions, and a third person (GT or PCG) was involved as arbitrator when needed. Full-text versions of studies deemed potentially relevant were retrieved and screened against inclusion criteria. The second reviewer independently screened 13% full-text papers. Authors were contacted when full-texts were not available. If authors did not reply, the paper was excluded.

### Quality assessment

The methodological quality of the included studies was assessed using the Mixed Methods Appraisal Tool (MMAT) (Hong *et al*., 2018). MMAT has two overall screening criteria and five criteria for each study design. One point was awarded for meeting the criteria indicators for each design-specific scoring domain. If papers mentioned some but not all of the criteria indicators 0.5 points were awarded (Gronholm *et al*., 2017). The MMAT score was used to determine the quality level of each paper, adapting the approach by Clay *et al*. (2020). Included studies were assessed for quality by the lead author, 33% of those were assessed by another reviewer to assess for consistency. Studies were not excluded based on methodological quality.

### Data extraction and analysis

A worksheet was developed to extract data from included papers (see online Supplementary materials). The classification of stigma by Pescosolido and Martin (2015) was used for study characteristics. It included courtesy, public, provider-based, structural and self-stigma. Missing data or elaboration on the interventions was requested from original authors.

Ineffective interventions were described alongside effective interventions for the study characteristics section of results; however, studies that did not report an effect on the outcomes were considered separately for the categorisation of ISC interventions. In the comparison of categories section, ineffective interventions were included in the synthesis to compare any differences between categories and effective and ineffective interventions.

The method of synthesising information on ISC interventions was based on thematic synthesis (Thomas and Harden, 2008). Literal descriptions of how the interventions were conducted and what the process entailed were extracted and coded line-by-line according to the upcoming meanings, content or themes. No pre-existing framework was used; thus, broad themes came from the descriptions provided. Due to the exploratory nature of this review, a general narrative synthesis was used to synthesise and describe the findings.

## Results

### Search results

**Fig. 1.**
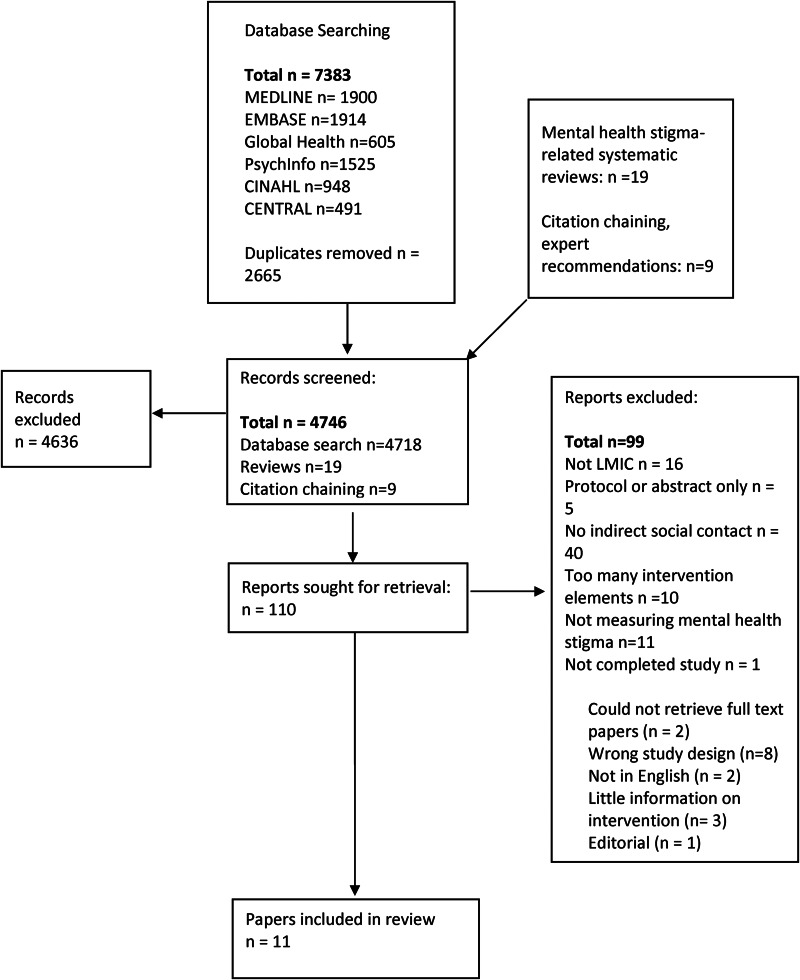
PRISMA flow diagram of selection of papers.

Of 7383 screened records, 11 papers (nine studies) were eligible for inclusion (Fig. 1). On two occasions, two papers referred to the same studies but with different follow-up points or different focus of outcome measures. These papers were considered jointly as reflective of one intervention study. Overall, 3630 participants were recruited through nine studies including healthcare workers, community leaders, families and members of the general population.

### Study characteristics

The main characteristics of the included studies are reported in Table 1.

**Table 1.** Key characteristics of included studies

Author (year)	Country	Study design	Population	Condition	Stigma	*N*	Contact medium	Detail of contact	Strategy mix	Delivery agent	% complete data	Duration	Assessment points	Stigma-related outcomes
Arthur *et al*. (2020*a*, 2020*b*)	Ghana	RCT	CL	Depression and schizophrenia	Public	140	V + PSV	Recovery + real life experiences	C + E	PWLE, caregivers (in vignette)	90	3 h	Pre, 12 weeks	Significant results for attitudes to mental illness (not social restrictiveness), non-significant for knowledge of depression
Fernandez *et al*. (2016)	Malaysia	RCT	HCW	Severe mental illness	Provider	102	V	Personal experience, recovery	C + E	PWLE, caregiver, health provider	100	40 min	Pre, 1 month	Significant effect for attitudes and behaviour
Finkelstein *et al*. (2008)	Russia	RCT	HCW	General mental illness	Provider	193	Stories in CAP	Personal experience, struggles, recovery, historical facts about poor treatment of people with MH problems	C + E	–	79	No information	Pre, post, 6 months	Significant effect for knowledge, attitudes and behaviour
Gürbüz *et al*. (2020)	Turkey	RCT	GP	OCD	Public	197	V	Symptoms, difficulties, social stigma, family member speaks about their experiences and reactions	C + E	PWLE, caregiver, psychiatrist	50.2	No information	Pre,2 weeks, 6 months	Significant effect for social distance and beliefs
Maulik *et al*. (2017, 2019)	India	Pre-post	GP	General mental illness	Public	1576	V + performance	Personal experience, domestic violence, suffering, the need for getting treatment	C + E	PWLE, caregivers, local theatre	73	N/A	Pre, post, 24 months	Post: significant for attitude, behaviour, 24 months: significant effect
Ng *et al*. (2017)	Malaysia	Pre-post	HCW	General mental illness	Provider	206	V	Myths, personal life, struggle, recovery, extended contact	C + E	PWLE, celebrity, nurse, lay person	99	4 min, 30 s	Pre, post	Significant effect for knowledge, attitudes and behaviour
Potts and Henderson (2021)	Ghana and Kenya	Pre-post	GP	General mental illness	Public	813	V + radio interview	First symptom, diagnosis, public and family stigma, treatment, recovery, how society should treat PWLE	C + E	PWLE	No information	No information	Pre, 1 week	Mixed effects: Kenya – significant change in knowledge, not attitude, behaviour; Ghana – significant change in social distance
Tergesen *et al*. (2021)	Nepal	Pilot RCT/RCT	HCW	Depression/depression, psychosis	Provider	100/213	V	Symptom, seeking treatment, tools PWLE learns, recovery	C	PWLE + caregiver	94, 96.8	8 min	Pre, post	Mixed results: PRCT: significant change in attitudes and knowledge; RCT: no effect
Vaghee *et al*. (2015)	Iran	RCT	Families	Schizophrenia	Public	90	V	Dark days, acceptance, treatment, coping skills, recovery	C	Caregivers	93, 75	4 h, 2 sessions	Pre, 1 month	Significant results for internalised stigma

RCT, randomised controlled trial; HCW, healthcare worker; GP, general population; CL, community leaders; V, video; PSV, problem-solving vignette; CAP, computer-assisted programme; PWLE, people with lived experience; C, contact; E, education; PRCT, pilot randomised controlled trial.

The quality of studies measured using MMAT varied (Table 2), with five high-quality papers fulfilling 80–90% of criteria, three moderate-quality papers fulfilling 60–80% of criteria and one of low quality fulfilling 40–50% of criteria.

**Table 2.** Summary of the study quality evaluated with MMAT

% MMAT criteria fulfilled	*N* (studies)	%	Overall quality
0–10	0	0	Very poor
10–20	0	0	
20–30	0	0	Poor
30–40	0	0	
40–50	1	11.1	
50–60	0	0	
60–70	1	11.1	Moderate
70–80	2	22.2	
80–90	5	55.5	High
90–100	0	0	

Five studies (55.6%) had a significant effect on all stigma-related outcomes, four had mixed results with small or medium effects and one randomised controlled trial (RCT) reported alongside a pilot RCT showed no significant effect. Studies included self-reported measures related to stigma that mainly included knowledge, attitudes or behaviour as proxy measures to evaluate changes in mental health-related stigma. These proxy measures were in accordance to the conceptualisation of stigma as issues of knowledge, attitudes and behaviour (Corrigan *et al*., 2012; Thornicroft *et al*., 2016).

### ISC intervention categorisation

#### Medium of delivery and points of ISC

All but one intervention (*n* = 8) used video-based media on its own or as one of the points of ISC. Videos varied greatly in their duration, ranging from 3 to 40 min. The majority of interventions (*n* = 5) showed the videos only at one point.

Three studies used multiple channels to deliver ISC. The Time to Change Global campaign in Kenya and Ghana (Potts and Henderson, 2021) used multiple types of ISC by using social media for videos and radio to broadcast interviews with local mental health champions. A campaign in India (Maulik *et al*., 2017, 2019) engaged the public with ISC through posters and local theatre play about people with mental health needs. A study in Ghana (Arthur *et al*., 2020*a*, 2020*b*) used video-based contact and a problem-solving exercise about a person with mental health needs where participants had to come up with a solution for recovery.

The only intervention that did not include videos as its method of ISC was conducted in Russia (Finkelstein *et al*., 2008) with special education university students. This RCT looked at the effectiveness of the computer-assisted education system that had education and contact strategies. Contact was in the form of stories that would appear in the computer-assisted education system with follow-up questions.

#### Content and main themes

Only broad themes could be extracted from the information in the papers. Two studies (Ng *et al*., 2017; Potts and Henderson, 2021) provided working links where videos could be accessed.

Seven interventions (Finkelstein *et al*., 2008; Vaghee *et al*., 2015; Fernandez *et al*., 2016; Maulik *et al*., 2017, 2019; Ng *et al*., 2017; Arthur *et al*., 2020*b*; Potts and Henderson, 2021) mentioned that during the videos or stories, person with lived experience described their personal experience of mental health needs. These experiences covered either mental health journeys, experiences with stigma or both. Information about caregiver videos mentioned personal experiences and reactions to the news about their family member having mental health problems, but further descriptions of the content were very limited.

Another commonly occurring theme was the use of a recovery story and seeking treatment (Finkelstein *et al*., 2008; Vaghee *et al*., 2015; Fernandez *et al*., 2016; Ng *et al*., 2017; Arthur *et al*., 2020*a*, 2020*b*; Potts and Henderson, 2021; Tergesen *et al*., 2021). Recovery was highlighted by people with lived experience, people in contact with them (caregivers or co-workers) or through a problem-solving exercise based on a vignette story. Treatment themes broadly covered the treatment options, the process and results of treatment or encouragement to seek treatment.

The study from Russia (Finkelstein *et al*., 2008) had a different approach of providing a personal story and recovery of a real-life person along historical facts and stories about negative treatment of people with mental health needs.

Another prominent theme on intervention effects was the presence of an emotional or empathetic response. The majority of effective or partly effective studies (Finkelstein *et al*., 2008; Vaghee *et al*., 2015; Maulik *et al*., 2017, 2019; Arthur *et al*., 2020*a*, 2020*b*; Potts and Henderson, 2021) mentioned that participants reported an emotional response towards the person with lived experience, or that the intervention aimed to elicit emotions from participants through ISC. The intervention conducted in Iran for families of service users (Vaghee *et al*., 2015) resulted in a discussion after the caregiver video during which people connected to experiences emotionally. In a study conducted in Russia (Finkelstein *et al*., 2008) qualitative data from students indicated that stories were an important part of the intervention. Qualitative results of a campaign in India (Maulik *et al*., 2017) revealed that people felt that they could relate and better understand the challenges faced by service users through theatrical performance and videos.

#### Combinations of interventions

The majority of studies (*n* = 7) used psychoeducation and ISC strategies to reduce stigma. The types of psychoeducation alongside ISC included delivery mediums such as presentations (Arthur *et al*., 2020*a*, 2020*b*), lectures (Fernandez *et al*., 2016), videos (Ng *et al*., 2017; Gürbüz *et al*., 2020), educational messages (Finkelstein *et al*., 2008), social media advertisements (Potts and Henderson, 2021) and printed materials such as posters and pamphlets (Maulik *et al*., 2017, 2019). One intervention compared the difference between direct social contact and education *v.* ISC (video) and education (Fernandez *et al*., 2016). Only one study in Iran used ISC intervention without combining it with another stigma-reducing strategy; this study compared ISC-only (video) and non-ISC interventions (psychoeducation and control) (Vaghee *et al*., 2015).

#### Delivery agent and interaction

In terms of delivery agents, the majority of interventions had people with lived experience as a delivery agent (Fernandez *et al*., 2016; Maulik *et al*., 2017, 2019; Ng *et al*., 2017; Gürbüz *et al*., 2020; Arthur *et al*., 2020*a*, 2020*b*; Potts and Henderson, 2021). One intervention (Vaghee *et al*., 2015) targeting families of patients with schizophrenia had only a caregiver as a delivery agent. Some interventions included people with lived experience and other key stakeholders as delivery agents; namely family members or caregivers (Fernandez *et al*., 2016; Maulik *et al*., 2017, 2019; Gürbüz *et al*., 2020; Arthur *et al*., 2020*a*, 2020*b*), healthcare workers (Fernandez *et al*., 2016; Ng *et al*., 2017) and celebrities known to have a mental health disorder, and a lay person talking about her co-worker with mental healthcare needs (Ng *et al*., 2017). Two interventions did not have a delivery agent at all or in one of the components of indirect contact (Finkelstein *et al*., 2008; Arthur *et al*., 2020*a*, 2020*b*) as the ISC was presented as a story in a computer program or as a vignette with a problem-solving exercise.

Another characteristic of ISC interventions in LMICs related to active and passive interaction with content. Active engagement entailed discussions in groups after watching videos (Vaghee *et al*., 2015), problem-solving exercises based on a vignette (Arthur *et al*., 2020*a*, 2020*b*) or responding to questions (Finkelstein *et al*., 2008). Passive contact occurred when participants would watch a video or listen to a radio programme without subsequently actively engaging with one's attitudes or knowledge either through interactive exercises or group discussions (Fernandez *et al*., 2016; Ng *et al*., 2017; Gürbüz *et al*., 2020; Potts and Henderson, 2021).

### Comparison and effectiveness of interventions with ISC

#### Content and main themes

The studies explicitly mentioning the cultural relevance of interventions showed that their positive results were sustained after a month (Vaghee *et al*., 2015), and even after 2 years (Maulik *et al*., 2019). Other effective interventions also had videos that either matched the local language, or that included local people with lived experience in the videos (Ng *et al*., 2017; Potts and Henderson, 2021).

The study in Ghana looking at depression and schizophrenia (Arthur *et al*., 2020*a*, 2020*b*) produced no significant changes in knowledge related to depression, and only some subscales (attitudes, beliefs) had significant differences at follow-up (Arthur *et al*., 2020*b*). Notably, the ineffective study (Tergesen *et al*., 2021) also targeted two conditions (depression and psychosis). Authors theorised that different stigma reduction strategies should be developed for different types of mental illnesses, or that more severe mental illnesses should not be paired in interventions with other mental health needs.

#### Intervention strategy comparison

There was a limited number of interventions that used only ISC. From those that did one found no significant results (Tergesen *et al*., 2021) and another (Vaghee *et al*., 2015) found significant changes in comparison to the control group for all subscales of Internalised Stigma of Mental Illness Scale (Ritsher *et al*., 2003). However, when compared to the psychoeducation group, significant changes were only detected for scores on the ‘social withdrawal’ and ‘discrimination experience’ subscales of the Internalised Stigma of Mental Illness Scale. Among the studies that used two strategies of education and contact (*n* = 7), four had significant effect on all the subscales of the stigma-related outcomes they used (Finkelstein *et al*., 2008; Fernandez *et al*., 2016; Ng *et al*., 2017; Gürbüz *et al*., 2020), and the rest had mixed results (Maulik *et al*., 2017; Arthur *et al*., 2020*a*, 2020*b*; Potts and Henderson, 2021).

As for the only ineffective study (Tergesen *et al*., 2021), no improvements were observed for implicit or explicit attitudes or diagnostic accuracy among medical students between the service user video (where service users with depression or schizophrenia shared their personal experience and recovery story) and a didactic video (healthcare provider talking about the treatment process).

#### Active and passive interaction

When it comes to the effectiveness of active and passive interaction, of four studies with active engagement two had a significant effect on most scores of knowledge, attitudes and behaviour at a 2-year follow-up (Maulik *et al*., 2017, 2019), and one had significant effects on stigma and knowledge scores after 6 months (Finkelstein *et al*., 2008). Two other studies showed partial improvement in attitudes (Arthur *et al*., 2020*b*), discrimination and social withdrawal (Vaghee *et al*., 2015).

Regarding studies with passive interaction with ISC, two produced significant positive effects on attitudes (Ng *et al*., 2017), social distance and help-seeking (Fernandez *et al*., 2016). Partial effectiveness was shown for knowledge or attitudes only (Potts and Henderson, 2021). The ineffective study (Tergesen *et al*., 2021) also involved passive interaction with service user videos.

## Discussion

This review aimed to categorise, compare and define ISC interventions to reduce mental health-related stigma in LMICs. Most included studies of ISC interventions were shown to be effective in reducing stigma either on all measures or certain subscales of measures. Currently limited evidence exists on interventions using only ISC to reduce stigma (Vaghee *et al*., 2015; Tergesen *et al*., 2021); more often ISC is paired with educational strategies (Finkelstein *et al*., 2008; Fernandez *et al*., 2016; Maulik *et al*., 2017, 2019; Ng *et al*., 2017; Arthur *et al*., 2020*a*, 2020*b*; Potts and Henderson, 2021). Existing evidence of higher effectiveness of combining education and contact strategies (Rüsch *et al*., 2005; Patten *et al*., 2012) was the rationale behind combining ISC with education. The included studies were all relatively recent, indicating a growing interest in this area. This review provides an important contribution through synthesising what is known to date about ISC interventions to reduce mental health-related stigma.

The most popular medium of ISC was videos, and indeed, previous reviews have shown that video-based contact can be effective in reducing stigma (Janoušková *et al*., 2017). Interestingly, there was only one intervention (Maulik *et al*., 2017, 2019) that used a creative outlet such as theatrical performance. Examples of using creative means of ISC from high-income countries (Michalak *et al*., 2014; Kosyluk *et al*., 2021) show significant changes in stigma which potentially indicates that such an approach can also be effective in LMICs.

As for the active and passive engagement with ISC, active interaction might contribute to more favourable results, and qualitative data found ISC (Maulik *et al*., 2017) being received as the most effective component of the intervention. However, it is difficult to judge as passive and active interactions produced both significant and mixed results.

### Content and culture

Some interventions provided limited details about the content of the ISC intervention (Finkelstein *et al*., 2008). This poses problems for future replicability and development of interventions. The content of ISC interventions is comparable with the content of direct contact interventions, where some of the strategies might include people sharing personal experiences, recovery stories or caregiver experience (Clement *et al*., 2012; Gonçalves *et al*., 2015). Sharing personal and recovery stories and experiences seems to be an important critical active ingredient for ISC in LMICs, as one or both of these themes have occurred in all interventions.

Cultural adaptation is likely to be important to produce effective and appropriate interventions for local communities. Given countries and cultures have differing sources of information that are considered appropriate or reliable (Semrau *et al*., 2015), it is crucial for interventions to adapt how and through whom stigma-reducing messages are translated. For instance, video contact by a peer caregiver in Iran (Vaghee *et al*., 2015) helped to create an environment where other families could safely share their experiences. Authors specified that differences between the individualistic, Western, and collectivist, Eastern, cultures were important considerations, and there was a need to arrange a safe environment for families to discuss and self-expose beliefs and experiences. Another example from the study conducted in India by Maulik *et al*. (2017) who purposefully developed a culturally relevant intervention stated that ‘Many participants mentioned that the drama and videos made them realize that they should not desert or abuse persons suffering from psychological problem, rather provide support to them.’ This further emphasises the importance and role of intervention's relevance and acceptability to the local culture.

### Defining ISC interventions in LMICs

After analysing the descriptions in the included papers, the following broad themes related to ISC interventions appeared: content, delivery agent, emotional response and effect on participants, cultural relevance or adaptation, interaction, delivery medium and effect on stigma. Given these broad themes we propose the following new definition of ISC in LMICs.

Indirect social contact entails a culturally/locally relevant active or passive interaction with real-life (or based on real-life) stories, narratives, or experiences of people with lived experience or those in contact or close to them (family or practitioners); and, uses online, technological, printed or other forms of traditional or new media for conveying information that elicits positive emotional or empathic responses.

### Strengths and limitations of the review

This is the first systematic review examining ISC in LMICs, providing a categorisation of these studies and a novel definition of ICS interventions in LMICs. This is in contrast to previous reviews that have focused on subtypes of ISC (Ando *et al*., 2011; Clement *et al*., 2013; Janoušková *et al*., 2017) and included studies from both HICs and LMICs, or considered different types of contact intervention strategies together (Clay *et al*., 2020; Hartog *et al*., 2020; Kaur *et al*., 2020).

This work does, however, need to be considered in view of some limitations. The included studies varied greatly in the level of detail of intervention descriptions, which could lead to results being more reliant on some papers than others for categorisation and definition. However, to mitigate such impacts authors were contacted for added details and all relevant information was extracted to capture main ideas and themes about ISC of each included study. Also, as is common in much stigma research, nearly all effectiveness measures were self-reported which may increase social desirability bias, and not all studies reported if the measures were validated in the local context.

### Implications and recommendations

A broader array of mediums and types of ISC interventions are seen in HICs compared to LMICs (Clement *et al*., 2013; Mehta *et al*., 2015; Thornicroft *et al*., 2016; Janoušková *et al*., 2017; Morgan *et al*., 2018; Rao *et al*., 2019). More studies are needed to explore if these might be appropriate in LMICs while addressing culture and local relevance. Given many difficulties of providing in-person contact it is important to continue investigating the effectiveness of ISC interventions on their own or together with other stigma-reducing strategies.

When reporting on ICS interventions, more details need to be provided on intervention components and content to facilitate further refinement of the ICS definition and categories in LMICs. Such insights will support the development of more effective ICS interventions.

More research evidence is needed from different regions, particularly low-income countries, as the current evidence-base is dominated by a small number of countries. Studies examining the long-term effectiveness of ICS interventions are also lacking.

## Conclusions

Based on current evidence from LMICs ISC can be categorised by content, combination of strategies, medium of delivery, delivery agent, condition and active/passive interaction. The most common way of delivering ISC was through video, but alternative ISC strategies were also effective. All interventions included recovery or personal experience, which seems to be an important part of ISC in LMICs.

ISC, specifically when paired with education strategies, is an effective approach for reducing mental health-related stigma in LMICs among the general population, healthcare workers and community leaders. At the moment, interventions with only ISC showed mixed or no significant changes in stigma. Active and passive interaction of participants with ISC needs to be explored further to reach more conclusive evidence. Thus, there is currently no conclusive evidence regarding the association between ICS intervention duration and effectiveness.

Our proposed definition of ISC can be refined further through consistency and clarity of future research.

## Data Availability

For access to data supporting this study, please email Akerke Makhmud: akerke.1.makhmud@kcl.ac.uk or akerkemakhmud@gmail.com.

## Supplementary material

For supplementary material accompanying this paper visit https://doi.org/10.1017/S2045796022000622. click here to view supplementary material
