# Supplementary material for: Indirect social contact interventions to reduce mental health-related stigma in low- and middle-income countries: systematic review
Source: Epidemiol Psychiatr Sci. 2022 Nov 9;31:e79. doi: 10.1017/S2045796022000622 (PMC9677443; doi:10.1017/S2045796022000622)
Supplement: Supplementary file 1 [file S2045796022000622sup001.docx]

Online Supplementary Materials

### Search Strategies for Medline, PsychInfo, EMBASE, Global Health, CINAHL, CENTRAL

**Ovid MEDLINE(R) ALL <1946 to June 29, 2021>**

1. mental health.ti,ab,kf.
2. Mental Health/
3. exp Mental Disorders/
4. mental disorder*.ti,ab,kf.
5. Mentally Ill Persons/
6. mental* ill*.ti,ab,kf.
7. Discrimination, Psychological/
8. social discriminat*.ti,ab,kf.
9. Prejudice/
10. prejudice.ti,ab,kf.
11. Social Marginalization/
12. social marginali*.ti,ab,kf.
13. Social Discrimination/
14. Social Isolation/
15. Social Stigma/
16. stigma*.ti,ab,kf.
17. Stereotyping/
18. stereotyp*.ti,ab,kf.
19. Attitude/
20. attitud*.ti,ab,kf.
21. "Attitude of Health Personnel"/
22. social acceptance.ti,ab,kf.
23. social perception.ti,ab,kf.
24. social exclusion.ti,ab,kf.
25. (afghanistan or albania or algeria or american samoa or angola or "antigua and barbuda" or antigua or barbuda or argentina or armenia or armenian or aruba or azerbaijan or bahrain or bangladesh or barbados or republic of belarus or belarus or byelarus or belorussia or byelorussian or belize or british honduras or benin or dahomey or bhutan or bolivia or "bosnia and herzegovina" or bosnia or herzegovina or botswana or bechuanaland or brazil or brasil or bulgaria or burkina faso or burkina fasso or upper volta or burundi or urundi or cabo verde or cape verde or cambodia or kampuchea or khmer republic or cameroon or cameron or cameroun or central african republic or ubangi shari or chad or chile or china or colombia or comoros or comoro islands or iles comores or mayotte or democratic republic of the congo or democratic republic congo or congo or zaire or costa rica or "cote d’ivoire" or "cote d’ ivoire" or cote divoire or cote d ivoire or ivory coast or croatia or cuba or cyprus or czech republic or czechoslovakia or djibouti or french somaliland or dominica or dominican republic or ecuador or egypt or united arab republic or el salvador or equatorial guinea or spanish guinea or eritrea or estonia or eswatini or swaziland or ethiopia or fiji or gabon or gabonese republic or gambia or "georgia (republic)" or georgian or ghana or gold coast or gibraltar or greece or grenada or guam or guatemala or guinea or guinea bissau or guyana or british guiana or haiti or hispaniola or honduras or hungary or india or indonesia or timor or iran or iraq or isle of man or jamaica or jordan or kazakhstan or kazakh or kenya or "democratic people’s republic of korea" or republic of korea or north korea or south korea or korea or kosovo or kyrgyzstan or kirghizia or kirgizstan or kyrgyz republic or kirghiz or laos or lao pdr or "lao people's democratic republic" or latvia or lebanon or lebanese republic or lesotho or basutoland or liberia or libya or libyan arab jamahiriya or lithuania or macau or macao or republic of north macedonia or macedonia or madagascar or malagasy republic or malawi or nyasaland or malaysia or malay federation or malaya federation or maldives or indian ocean islands or indian ocean or mali or malta or micronesia or federated states of micronesia or kiribati or marshall islands or nauru or northern mariana islands or palau or tuvalu or mauritania or mauritius or mexico or moldova or moldovian or mongolia or montenegro or morocco or ifni or mozambique or portuguese east africa or myanmar or burma or namibia or nepal or netherlands antilles or nicaragua or niger or nigeria or oman or muscat or pakistan or panama or papua new guinea or new guinea or paraguay or peru or philippines or philipines or phillipines or phillippines or poland or "polish people's republic" or portugal or portuguese republic or puerto rico or romania or russia or russian federation or ussr or soviet union or union of soviet socialist republics or rwanda or ruanda or samoa or pacific islands or polynesia or samoan islands or navigator island or navigator islands or "sao tome and principe" or saudi arabia or senegal or serbia or seychelles or sierra leone or slovakia or slovak republic or slovenia or melanesia or solomon island or solomon islands or norfolk island or norfolk islands or somalia or south africa or south sudan or sri lanka or ceylon or "saint kitts and nevis" or "st. kitts and nevis" or saint lucia or "st. lucia" or "saint vincent and the grenadines" or saint vincent or "st. vincent" or grenadines or sudan or suriname or surinam or dutch guiana or netherlands guiana or syria or syrian arab republic or tajikistan or tadjikistan or tadzhikistan or tadzhik or tanzania or tanganyika or thailand or siam or timor leste or east timor or togo or togolese republic or tonga or "trinidad and tobago" or trinidad or tobago or tunisia or turkey or turkmenistan or turkmen or uganda or ukraine or uruguay or uzbekistan or uzbek or vanuatu or new hebrides or venezuela or vietnam or viet nam or middle east or west bank or gaza or palestine or yemen or yugoslavia or zambia or zimbabwe or northern rhodesia or global south or africa south of the sahara or sub-saharan africa or subsaharan africa or africa, central or central africa or africa, northern or north africa or northern africa or magreb or maghrib or sahara or africa, southern or southern africa or africa, eastern or east africa or eastern africa or africa, western or west africa or western africa or west indies or indian ocean islands or caribbean or central america or latin america or "south and central america" or south america or asia, central or central asia or asia, northern or north asia or northern asia or asia, southeastern or southeastern asia or south eastern asia or southeast asia or south east asia or asia, western or western asia or europe, eastern or east europe or eastern europe or developing country or developing countries or developing nation? or developing population? or developing world or less developed countr* or less developed nation? or less developed population? or less developed world or lesser developed countr* or lesser developed nation? or lesser developed population? or lesser developed world or under developed countr* or under developed nation? or under developed population? or under developed world or underdeveloped countr* or underdeveloped nation? or underdeveloped population? or underdeveloped world or middle income countr* or middle income nation? or middle income population? or low income countr* or low income nation? or low income population? or lower income countr* or lower income nation? or lower income population? or underserved countr* or underserved nation? or underserved population? or underserved world or under served countr* or under served nation? or under served population? or under served world or deprived countr* or deprived nation? or deprived population? or deprived world or poor countr* or poor nation? or poor population? or poor world or poorer countr* or poorer nation? or poorer population? or poorer world or developing econom* or less developed econom* or lesser developed econom* or under developed econom* or underdeveloped econom* or middle income econom* or low income econom* or lower income econom* or low gdp or low gnp or low gross domestic or low gross national or lower gdp or lower gnp or lower gross domestic or lower gross national or lmic or lmics or third world or lami countr* or transitional countr* or emerging economies or emerging nation?).ti,ab,sh,kf.
26. (afghan or afghans or afghani or albanian? algerian? or american samoan? or angolan? or antiguan? or barbudan? or argentine? or argentinian? or argentinean? or armenian? or aruban? or azerbaijani? or bahraini? or bangladeshi? or bangalees or bajan? or belarusian? or byelorussian? or belizean? or beninese? or bhutanese or bolivian? or bosnian? or botswana or batswana or brazilian? or brasilian? or bulgarian? or burkinabe or burkinese or burundian? or cape verdean? or cabo verdean? or cambodian? or khmer or cameroonian? or central african? or chadian? or chilean? or chinese or colombian? or comorian? or congolese or costa rican? or ivorian? or croatian? or cuban? or cypriot? or czech? or djiboutian? or dominican? or ecuadorian? or egyptian? or salvadoran? or equatorial guinean? or equatoguinean? or eritrean? or estonian? or swazi? or swati? or ethiopian? or fijian or gabonese or gabonaise or gambian? or georgian? or ghanaian? or gibraltarian? or greek? or grenadian? or guamanian? or guatemalan? or guinean? or bissau guinean? or guyanese or haitian? or honduran? or hungarian? or indian? or indonesian? or iranian? or iraqian? or iraqi? or manx or jamaican? or jordanian? or kazakhstani? or kenyan? or kirabati or kirabatian? or north korean? or korean? or kosovar? or kosovan? or kyrgyz* or lao or laotian? or latvian? or lebanese or lesothan? or lesothonian? or mosotho or basotho or liberian? or libyan? or lithuanian? or macanese or macedonian? or malagasy or madagascan? or malawian? or malaysian? or maldivian? or malian? or maltese or marshallese? or mauritanian? or mauritian? or mexican? or micronesian? or moldovan? or mongolian? or mongol or montenegrin? or moroccan? or mozambican? or burmese or myanma or namibian? or nauruan? or nepali or nepalese or netherlands antillean? or nicaraguan? or nigerien? or nigerian? or northern mariana islander? or mariana? or omani? or pakistani? or palauan? or panamanian? or papua new guinean? or paraguayan? or peruvian? or philippine? or philipine? or phillipine? or phillippine? or filipino? or filipina? or polish or pole or poles or portuguese or puerto rican? or romanian? or russian? or soviet people or soviet population or rwandan? or rwandese or ruandan? or ruandese or samoan? or sao tomean? or santomean? or saudi arabian? or saudi? or senegalese or serbian? or montenegrin? or seychellois or seychelloise? or sierra leonean? or slovak? or slovene? or solomon islander? or somali? or south african? or south sudanese or sri lankan? or ceylonese or kittitian? or nevisian? or saint lucian? or vincentian? or sudanese or surinamese? or syrian? or tajik? or tajikistani? or tanzanian? or tanganyikan? or thai or timorese? or togolese or tongan? or trinidadian? or tobagonian? or tunisian? or turk? or turkish or turkmen? or tuvaluan? or ugandan? or ukrainian? or uruguayan? or uzbek? or vanuatu* or venezuelan? or vietnamese or yemeni? or yemenite? or yemenese or yugoslav? or yugoslavian? or zambian? or zimbabwean?).ti,ab,sh,kf.
27. Community Mental Health Services/
28. Evaluation Study/
29. Follow-Up Studies/
30. Program Evaluation/
31. Health Knowledge, Attitudes, Practice/
32. Randomized Controlled Trial/
33. Internet-Based Intervention/
34. Health Education/
35. clinical trial.ti,ab,kf.
36. (stigma* adj5 reduc*).ti,ab,kf.
37. (discrim* adj5 reduc*).ti,ab,kf.
38. campaign*.ti,ab,kf.
39. intervent*.ti,ab,kf.
40. ((improve* or change*) adj5 (knowledge* or attitude* or behavio*)).ti,ab,kf.
41. anti?stigma*.ti,ab,kf.
42. workshop*.ti,ab,kf.
43. (community adj5 "mental health").ti,ab,kf.
44. (change adj5 stigma).ti,ab,kf.
45. pre-post.ti,ab,kf.
46. evaluat*.ti,ab,kf.
47. Blogging/
48. Narration/
49. Personal Narrative/
50. Social Media/
51. Internet/
52. Video-Audio Media/
53. Multimedia/
54. storytel*.ti,ab,kf.
55. blog*.ti,ab,kf.
56. social media.ti,ab,kf.
57. internet.ti,ab,kf.
58. presentation.ti,ab,kf.
59. indirect contact.ti,ab,kf.
60. social contact.ti,ab,kf.
61. extended contact.ti,ab,kf.
62. imagin* contact.ti,ab,kf.
63. vicario* contact.ti,ab,kf.
64. narrativ*.ti,ab,kf.
65. film*.ti,ab,kf.
66. video.ti,ab,kf.
67. theatr*.ti,ab,kf.
68. photo?voice.ti,ab,kf.
69. engag*.ti,ab,kf.
70. psychoeducation.ti,ab,kf.
71. 1 or 2 or 3 or 4 or 5 or 6
72. 7 or 8 or 9 or 10 or 11 or 12 or 13 or 14 or 15 or 16 or 17 or 18 or 19 or 20 or 21 or 22 or 23 or 24 or 69
73. 25 or 26
74. 27 or 28 or 29 or 30 or 31 or 32 or 33 or 34 or 35 or 36 or 37 or 38 or 39 or 40 or 41 or 42 or 43 or 44 or 45 or 46 or 70
75. 27 or 38 or 42 or 43 or 47 or 48 or 49 or 50 or 51 or 52 or 53 or 54 or 55 or 56 or 57 or 58 or 59 or 60 or 61 or 62 or 63 or 64 or 65 or 66 or 67 or 68 or 69
76. 71 and 72 and 73 and 74 and 75
77. limit 76 to (english language and humans)

**APA PsycInfo <1806 to June Week 2 2021>**

1. mental health.ti,ab,id.
2. mental health.ti,ab,id.
3. Mental Health/
4. exp Mental Disorders/
5. mental disorder*.ti,ab,id.
6. mental* ill*.ti,ab,id.
7. social discriminat*.ti,ab,id.
8. Prejudice/
9. prejudice.ti,ab,id.
10. social marginali*.ti,ab,id.
11. Social Discrimination/
12. Social Isolation/
13. Social Stigma/
14. stigma*.ti,ab,id.
15. Stereotyping/
16. stereotyp*.ti,ab,id.
17. attitud*.ti,ab,id.
18. social acceptance.ti,ab,id.
19. social perception.ti,ab,id.
20. social exclusion.ti,ab,id.
21. (afghanistan or albania or algeria or american samoa or angola or "antigua and barbuda" or antigua or barbuda or argentina or armenia or armenian or aruba or azerbaijan or bahrain or bangladesh or barbados or republic of belarus or belarus or byelarus or belorussia or byelorussian or belize or british honduras or benin or dahomey or bhutan or bolivia or "bosnia and herzegovina" or bosnia or herzegovina or botswana or bechuanaland or brazil or brasil or bulgaria or burkina faso or burkina fasso or upper volta or burundi or urundi or cabo verde or cape verde or cambodia or kampuchea or khmer republic or cameroon or cameron or cameroun or central african republic or ubangi shari or chad or chile or china or colombia or comoros or comoro islands or iles comores or mayotte or democratic republic of the congo or democratic republic congo or congo or zaire or costa rica or "cote d’ivoire" or "cote d’ ivoire" or cote divoire or cote d ivoire or ivory coast or croatia or cuba or cyprus or czech republic or czechoslovakia or djibouti or french somaliland or dominica or dominican republic or ecuador or egypt or united arab republic or el salvador or equatorial guinea or spanish guinea or eritrea or estonia or eswatini or swaziland or ethiopia or fiji or gabon or gabonese republic or gambia or "georgia (republic)" or georgian or ghana or gold coast or gibraltar or greece or grenada or guam or guatemala or guinea or guinea bissau or guyana or british guiana or haiti or hispaniola or honduras or hungary or india or indonesia or timor or iran or iraq or isle of man or jamaica or jordan or kazakhstan or kazakh or kenya or "democratic people’s republic of korea" or republic of korea or north korea or south korea or korea or kosovo or kyrgyzstan or kirghizia or kirgizstan or kyrgyz republic or kirghiz or laos or lao pdr or "lao people's democratic republic" or latvia or lebanon or lebanese republic or lesotho or basutoland or liberia or libya or libyan arab jamahiriya or lithuania or macau or macao or republic of north macedonia or macedonia or madagascar or malagasy republic or malawi or nyasaland or malaysia or malay federation or malaya federation or maldives or indian ocean islands or indian ocean or mali or malta or micronesia or federated states of micronesia or kiribati or marshall islands or nauru or northern mariana islands or palau or tuvalu or mauritania or mauritius or mexico or moldova or moldovian or mongolia or montenegro or morocco or ifni or mozambique or portuguese east africa or myanmar or burma or namibia or nepal or netherlands antilles or nicaragua or niger or nigeria or oman or muscat or pakistan or panama or papua new guinea or new guinea or paraguay or peru or philippines or philipines or phillipines or phillippines or poland or "polish people's republic" or portugal or portuguese republic or puerto rico or romania or russia or russian federation or ussr or soviet union or union of soviet socialist republics or rwanda or ruanda or samoa or pacific islands or polynesia or samoan islands or navigator island or navigator islands or "sao tome and principe" or saudi arabia or senegal or serbia or seychelles or sierra leone or slovakia or slovak republic or slovenia or melanesia or solomon island or solomon islands or norfolk island or norfolk islands or somalia or south africa or south sudan or sri lanka or ceylon or "saint kitts and nevis" or "st. kitts and nevis" or saint lucia or "st. lucia" or "saint vincent and the grenadines" or saint vincent or "st. vincent" or grenadines or sudan or suriname or surinam or dutch guiana or netherlands guiana or syria or syrian arab republic or tajikistan or tadjikistan or tadzhikistan or tadzhik or tanzania or tanganyika or thailand or siam or timor leste or east timor or togo or togolese republic or tonga or "trinidad and tobago" or trinidad or tobago or tunisia or turkey or turkmenistan or turkmen or uganda or ukraine or uruguay or uzbekistan or uzbek or vanuatu or new hebrides or venezuela or vietnam or viet nam or middle east or west bank or gaza or palestine or yemen or yugoslavia or zambia or zimbabwe or northern rhodesia or global south or africa south of the sahara or sub-saharan africa or subsaharan africa or africa, central or central africa or africa, northern or north africa or northern africa or magreb or maghrib or sahara or africa, southern or southern africa or africa, eastern or east africa or eastern africa or africa, western or west africa or western africa or west indies or indian ocean islands or caribbean or central america or latin america or "south and central america" or south america or asia, central or central asia or asia, northern or north asia or northern asia or asia, southeastern or southeastern asia or south eastern asia or southeast asia or south east asia or asia, western or western asia or europe, eastern or east europe or eastern europe or developing country or developing countries or developing nation? or developing population? or developing world or less developed countr* or less developed nation? or less developed population? or less developed world or lesser developed countr* or lesser developed nation? or lesser developed population? or lesser developed world or under developed countr* or under developed nation? or under developed population? or under developed world or underdeveloped countr* or underdeveloped nation? or underdeveloped population? or underdeveloped world or middle income countr* or middle income nation? or middle income population? or low income countr* or low income nation? or low income population? or lower income countr* or lower income nation? or lower income population? or underserved countr* or underserved nation? or underserved population? or underserved world or under served countr* or under served nation? or under served population? or under served world or deprived countr* or deprived nation? or deprived population? or deprived world or poor countr* or poor nation? or poor population? or poor world or poorer countr* or poorer nation? or poorer population? or poorer world or developing econom* or less developed econom* or lesser developed econom* or under developed econom* or underdeveloped econom* or middle income econom* or low income econom* or lower income econom* or low gdp or low gnp or low gross domestic or low gross national or lower gdp or lower gnp or lower gross domestic or lower gross national or lmic or lmics or third world or lami countr* or transitional countr* or emerging economies or emerging nation?).ti,ab,sh,id.
22. (afghan or afghans or afghani or albanian? algerian? or american samoan? or angolan? or antiguan? or barbudan? or argentine? or argentinian? or argentinean? or armenian? or aruban? or azerbaijani? or bahraini? or bangladeshi? or bangalees or bajan? or belarusian? or byelorussian? or belizean? or beninese? or bhutanese or bolivian? or bosnian? or botswana or batswana or brazilian? or brasilian? or bulgarian? or burkinabe or burkinese or burundian? or cape verdean? or cabo verdean? or cambodian? or khmer or cameroonian? or central african? or chadian? or chilean? or chinese or colombian? or comorian? or congolese or costa rican? or ivorian? or croatian? or cuban? or cypriot? or czech? or djiboutian? or dominican? or ecuadorian? or egyptian? or salvadoran? or equatorial guinean? or equatoguinean? or eritrean? or estonian? or swazi? or swati? or ethiopian? or fijian or gabonese or gabonaise or gambian? or georgian? or ghanaian? or gibraltarian? or greek? or grenadian? or guamanian? or guatemalan? or guinean? or bissau guinean? or guyanese or haitian? or honduran? or hungarian? or indian? or indonesian? or iranian? or iraqian? or iraqi? or manx or jamaican? or jordanian? or kazakhstani? or kenyan? or kirabati or kirabatian? or north korean? or korean? or kosovar? or kosovan? or kyrgyz* or lao or laotian? or latvian? or lebanese or lesothan? or lesothonian? or mosotho or basotho or liberian? or libyan? or lithuanian? or macanese or macedonian? or malagasy or madagascan? or malawian? or malaysian? or maldivian? or malian? or maltese or marshallese? or mauritanian? or mauritian? or mexican? or micronesian? or moldovan? or mongolian? or mongol or montenegrin? or moroccan? or mozambican? or burmese or myanma or namibian? or nauruan? or nepali or nepalese or netherlands antillean? or nicaraguan? or nigerien? or nigerian? or northern mariana islander? or mariana? or omani? or pakistani? or palauan? or panamanian? or papua new guinean? or paraguayan? or peruvian? or philippine? or philipine? or phillipine? or phillippine? or filipino? or filipina? or polish or pole or poles or portuguese or puerto rican? or romanian? or russian? or soviet people or soviet population or rwandan? or rwandese or ruandan? or ruandese or samoan? or sao tomean? or santomean? or saudi arabian? or saudi? or senegalese or serbian? or montenegrin? or seychellois or seychelloise? or sierra leonean? or slovak? or slovene? or solomon islander? or somali? or south african? or south sudanese or sri lankan? or ceylonese or kittitian? or nevisian? or saint lucian? or vincentian? or sudanese or surinamese? or syrian? or tajik? or tajikistani? or tanzanian? or tanganyikan? or thai or timorese? or togolese or tongan? or trinidadian? or tobagonian? or tunisian? or turk? or turkish or turkmen? or tuvaluan? or ugandan? or ukrainian? or uruguayan? or uzbek? or vanuatu* or venezuelan? or vietnamese or yemeni? or yemenite? or yemenese or yugoslav? or yugoslavian? or zambian? or zimbabwean?).ti,ab,sh,id.
23. Discrimination/
24. Marginalization/
25. Attitudes/
26. "Mental Illness (Attitudes Toward)"/
27. Mental Health Stigma/
28. Stigma/
29. Self-Stigma/
30. Community Mental Health Services/
31. Mental Health Program Evaluation/
32. Followup Studies/
33. Health Knowledge/
34. Randomized Controlled Trial/
35. Internet/
36. Health Education/
37. Psychoeducation/
38. clinical trial.ti,ab,id.
39. internet.ti,ab,id.
40. (stigma* adj5 reduc*).id,ti,ab.
41. (discrim* adj5 reduc*).ti,ab,id.
42. campaign*.ti,ab,id.
43. intervent*.ti,ab,id.
44. ((improv* or chang*) adj5 (knowledge* or attitude* or behavio*)).ti,ab,id.
45. anti?stigma.ti,ab,id.
46. workshop*.ti,ab,id.
47. (community adj5 "mental health").ti,ab,id.
48. (change adj5 stigma).ti,ab,id.
49. pre-post.ti,ab,id.
50. evaluat*.ti,ab,id.
51. Blog/
52. Narratives/
53. Storytelling/
54. Social Media/
55. Video-Based Interventions/
56. Digital Media/
57. Audiovisual Communications Media/
58. Mass Media/
59. Multimedia/
60. storytel*.ti,ab,id.
61. blog*.ti,ab,id.
62. social media.ti,ab,id.
63. internet.ti,ab,id.
64. presentation.ti,ab,id.
65. indirect contact.ti,ab,id.
66. social contact.ti,ab,id.
67. extended contact.ti,ab,id.
68. imagin* contact.ti,ab,id.
69. vicario* contact.ti,ab,id.
70. narrativ*.ti,ab,id.
71. film*.ti,ab,id.
72. video.ti,ab,id.
73. theatr*.ti,ab,id.
74. photo?voice.ti,ab,id.
75. engag*.ti,ab,id.
76. psychoeducation.ti,ab,id.
77. 1 or 2 or 3 or 4 or 5 or 6
78. 7 or 8 or 9 or 10 or 11 or 12 or 13 or 14 or 15 or 16 or 17 or 18 or 19 or 20 or 23 or 24 or 25 or 26 or 27 or 28 or 29 or 75
79. 21 or 22
80. 30 or 31 or 32 or 33 or 34 or 38 or 40 or 41 or 42 or 43 or 44 or 45 or 46 or 47 or 48 or 49 or 50
81. 30 or 35 or 36 or 37 or 39 or 42 or 46 or 47 or 51 or 52 or 53 or 54 or 55 or 56 or 57 or 58 or 59 or 60 or 61 or 62 or 63 or 64 or 65 or 66 or 67 or 68 or 69 or 70 or 71 or 72 or 73 or 74 or 75 or 76
82. 77 and 78 and 79 and 80 and 81
83. limit 82 to (human and english language)

**Embase <1974 to 2021 June 29>**

1. mental health.ti,ab,kw.
2. mental health/
3. mental disorder*.ti,ab,kw.
4. mental patient/
5. mental* ill*.ti,ab,kw.
6. social discrimination/
7. discriminat*.ti,ab,kw.
8. prejudice/
9. prejudice.ti,ab,kw.
10. social exclusion/
11. social marginali?ation.ti,ab,kw.
12. social isolation/
13. stigma/
14. social stigma/
15. stigma*.ti,ab,kw.
16. stereotyping/
17. stereotyp*.ti,ab,kw.
18. attitude/
19. social attitude/
20. attitude to mental illness/
21. attitud*.ti,ab,kw.
22. health personnel attitude/
23. social acceptance/
24. social acceptance.ti,ab,kw.
25. perception/
26. social perception.ti,ab,kw.
27. social exclusion.ti,ab,kw.
28. (afghanistan or albania or algeria or american samoa or angola or "antigua and barbuda" or antigua or barbuda or argentina or armenia or armenian or aruba or azerbaijan or bahrain or bangladesh or barbados or republic of belarus or belarus or byelarus or belorussia or byelorussian or belize or british honduras or benin or dahomey or bhutan or bolivia or "bosnia and herzegovina" or bosnia or herzegovina or botswana or bechuanaland or brazil or brasil or bulgaria or burkina faso or burkina fasso or upper volta or burundi or urundi or cabo verde or cape verde or cambodia or kampuchea or khmer republic or cameroon or cameron or cameroun or central african republic or ubangi shari or chad or chile or china or colombia or comoros or comoro islands or iles comores or mayotte or democratic republic of the congo or democratic republic congo or congo or zaire or costa rica or "cote d’ivoire" or "cote d’ ivoire" or cote divoire or cote d ivoire or ivory coast or croatia or cuba or cyprus or czech republic or czechoslovakia or djibouti or french somaliland or dominica or dominican republic or ecuador or egypt or united arab republic or el salvador or equatorial guinea or spanish guinea or eritrea or estonia or eswatini or swaziland or ethiopia or fiji or gabon or gabonese republic or gambia or "georgia (republic)" or georgian or ghana or gold coast or gibraltar or greece or grenada or guam or guatemala or guinea or guinea bissau or guyana or british guiana or haiti or hispaniola or honduras or hungary or india or indonesia or timor or iran or iraq or isle of man or jamaica or jordan or kazakhstan or kazakh or kenya or "democratic people’s republic of korea" or republic of korea or north korea or south korea or korea or kosovo or kyrgyzstan or kirghizia or kirgizstan or kyrgyz republic or kirghiz or laos or lao pdr or "lao people's democratic republic" or latvia or lebanon or lebanese republic or lesotho or basutoland or liberia or libya or libyan arab jamahiriya or lithuania or macau or macao or republic of north macedonia or macedonia or madagascar or malagasy republic or malawi or nyasaland or malaysia or malay federation or malaya federation or maldives or indian ocean islands or indian ocean or mali or malta or micronesia or federated states of micronesia or kiribati or marshall islands or nauru or northern mariana islands or palau or tuvalu or mauritania or mauritius or mexico or moldova or moldovian or mongolia or montenegro or "montenegro (republic)" or morocco or ifni or mozambique or portuguese east africa or myanmar or burma or namibia or nepal or netherlands antilles or nicaragua or niger or nigeria or oman or muscat or pakistan or panama or papua new guinea or new guinea or paraguay or peru or philippines or philipines or phillipines or phillippines or poland or "polish people's republic" or portugal or portuguese republic or puerto rico or romania or russia or russian federation or ussr or soviet union or union of soviet socialist republics or rwanda or ruanda or samoa or pacific islands or polynesia or samoan islands or navigator island or navigator islands or "sao tome and principe" or saudi arabia or senegal or serbia or seychelles or sierra leone or slovakia or slovak republic or slovenia or melanesia or solomon island or solomon islands or norfolk island or norfolk islands or somalia or south africa or south sudan or sri lanka or ceylon or "saint kitts and nevis" or "st. kitts and nevis" or saint lucia or "st. lucia" or "saint vincent and the grenadines" or saint vincent or "st. vincent" or grenadines or sudan or suriname or surinam or dutch guiana or netherlands guiana or syria or syrian arab republic or tajikistan or tadjikistan or tadzhikistan or tadzhik or tanzania or tanganyika or thailand or siam or timor leste or east timor or togo or togolese republic or tonga or "trinidad and tobago" or trinidad or tobago or tunisia or "turkey (republic)" or turkey or turkmenistan or turkmen or uganda or ukraine or uruguay or uzbekistan or uzbek or vanuatu or new hebrides or venezuela or vietnam or viet nam or middle east or west bank or gaza or palestine or yemen or yugoslavia or zambia or zimbabwe or northern rhodesia or global south or africa south of the sahara or "sub saharan africa" or subsaharan africa or africa, central or central africa or africa, northern or north africa or northern africa or magreb or maghrib or sahara or africa, southern or southern africa or africa, eastern or east africa or eastern africa or africa, western or west africa or western africa or west indies or indian ocean islands or caribbean region or caribbean islands or caribbean or central america or latin america or "south and central america" or south america or asia, central or central asia or asia, northern or north asia or northern asia or asia, southeastern or southeastern asia or south eastern asia or southeast asia or south east asia or asia, western or western asia or europe, eastern or east europe or eastern europe or developing country or developing countries or developing nation? or developing population? or developing world or less developed countr* or less developed nation? or less developed population? or less developed world or lesser developed countr* or lesser developed nation? or lesser developed population? or lesser developed world or under developed countr* or under developed nation? or under developed population? or under developed world or underdeveloped countr* or underdeveloped nation? or underdeveloped population? or underdeveloped world or middle income countr* or middle income nation? or middle income population? or low income countr* or low income nation? or low income population? or lower income countr* or lower income nation? or lower income population? or underserved countr* or underserved nation? or underserved population? or underserved world or under served countr* or under served nation? or under served population? or under served world or deprived countr* or deprived nation? or deprived population? or deprived world or poor countr* or poor nation? or poor population? or poor world or poorer countr* or poorer nation? or poorer population? or poorer world or developing econom* or less developed econom* or lesser developed econom* or under developed econom* or underdeveloped econom* or middle income econom* or low income econom* or lower income econom* or low gdp or low gnp or low gross domestic or low gross national or lower gdp or lower gnp or lower gross domestic or lower gross national or lmic or lmics or third world or lami countr* or transitional countr* or emerging economies or emerging nation?).ti,ab,sh,kw.
29. (afghan or afghans or afghani or albanian? algerian? or american samoan? or angolan? or antiguan? or barbudan? or argentine? or argentinian? or argentinean? or armenian? or aruban? or azerbaijani? or bahraini? or bangladeshi? or bangalees or bajan? or belarusian? or byelorussian? or belizean? or beninese? or bhutanese or bolivian? or bosnian? or botswana or batswana or brazilian? or brasilian? or bulgarian? or burkinabe or burkinese or burundian? or cape verdean? or cabo verdean? or cambodian? or khmer or cameroonian? or central african? or chadian? or chilean? or chinese or colombian? or comorian? or congolese or costa rican? or ivorian? or croatian? or cuban? or cypriot? or czech? or djiboutian? or dominican? or ecuadorian? or egyptian? or salvadoran? or equatorial guinean? or equatoguinean? or eritrean? or estonian? or swazi? or swati? or ethiopian? or fijian or gabonese or gabonaise or gambian? or georgian? or ghanaian? or gibraltarian? or greek? or grenadian? or guamanian? or guatemalan? or guinean? or bissau guinean? or guyanese or haitian? or honduran? or hungarian? or indian? or indonesian? or iranian? or iraqian? or iraqi? or manx or jamaican? or jordanian? or kazakhstani? or kenyan? or kirabati or kirabatian? or north korean? or korean? or kosovar? or kosovan? or kyrgyz* or lao or laotian? or latvian? or lebanese or lesothan? or lesothonian? or mosotho or basotho or liberian? or libyan? or lithuanian? or macanese or macedonian? or malagasy or madagascan? or malawian? or malaysian? or maldivian? or malian? or maltese or marshallese? or mauritanian? or mauritian? or mexican? or micronesian? or moldovan? or mongolian? or mongol or montenegrin? or moroccan? or mozambican? or burmese or myanma or namibian? or nauruan? or nepali or nepalese or netherlands antillean? or nicaraguan? or nigerien? or nigerian? or northern mariana islander? or mariana? or omani? or pakistani? or palauan? or panamanian? or papua new guinean? or paraguayan? or peruvian? or philippine? or philipine? or phillipine? or phillippine? or filipino? or filipina? or polish or pole or poles or portuguese or puerto rican? or romanian? or russian? or soviet people or soviet population or rwandan? or rwandese or ruandan? or ruandese or samoan? or sao tomean? or santomean? or saudi arabian? or saudi? or senegalese or serbian? or montenegrin? or seychellois or seychelloise? or sierra leonean? or slovak? or slovene? or solomon islander? or somali? or south african? or south sudanese or sri lankan? or ceylonese or kittitian? or nevisian? or saint lucian? or vincentian? or sudanese or surinamese? or syrian? or tajik? or tajikistani? or tanzanian? or tanganyikan? or thai or timorese? or togolese or tongan? or trinidadian? or tobagonian? or tunisian? or turk? or turkish or turkmen? or tuvaluan? or ugandan? or ukrainian? or uruguayan? or uzbek? or vanuatu* or venezuelan? or vietnamese or yemeni? or yemenite? or yemenese or yugoslav? or yugoslavian? or zambian? or zimbabwean?).ti,ab,sh,kw.
30. community mental health service/
31. evaluation study/
32. Program Evaluation/
33. randomized controlled trial/
34. web-based intervention/
35. follow up/
36. attitude to health/
37. health education/
38. psychoeducation/
39. clinical trial.ti,ab,kw.
40. (stigma* adj5 reduc*).ti,ab,kw.
41. (discrim* adj5 reduc*).ti,ab,kw.
42. campaign*.ti,ab,kw.
43. intervent*.ti,ab,kw.
44. intervention study/
45. ((improve* or change*) adj5 (knowledge* or attitude* or behavio*)).ti,ab,kw.
46. anti?stigma.ti,ab,kw.
47. workshop/
48. workshop*.ti,ab,kw.
49. (community adj5 "mental health").ti,ab,kw.
50. (change adj5 stigma).ti,ab,kw.
51. pre-post.ti,ab,kw.
52. evaluat*.ti,ab,kw.
53. blogging/
54. narrative/
55. social media/
56. mass medium/
57. videorecording/
58. audio recording/
59. multimedia/
60. storytelling/
61. storytel*.ti,ab,kw.
62. blog*.ti,ab,kw.
63. social media.ti,ab,kw.
64. internet.ti,ab,kw.
65. presentation.ti,ab,kw.
66. indirect contact.ti,ab,kw.
67. social contact.ti,ab,kw.
68. extended contact.ti,ab,kw.
69. imagin* contact.ti,ab,kw.
70. vicario* contact.ti,ab,kw.
71. narrativ*.ti,ab,kw.
72. film*.ti,ab,kw.
73. video.ti,ab,kw.
74. theatr*.ti,ab,kw.
75. photo?voice.ti,ab,kw.
76. engag*.ti,ab,kw.
77. psychoeducation.ti,ab,kw.
78. mental disease/
79. 1 or 2 or 3 or 4 or 5 or 78
80. 28 or 29
81. 6 or 7 or 8 or 9 or 10 or 11 or 12 or 13 or 14 or 15 or 16 or 17 or 18 or 19 or 20 or 21 or 22 or 23 or 24 or 25 or 26 or 27 or 76
82. 30 or 31 or 32 or 33 or 34 or 35 or 36 or 37 or 38 or 39 or 40 or 41 or 42 or 43 or 44 or 45 or 46 or 47 or 48 or 49 or 50 or 51 or 52 or 77
83. 30 or 42 or 47 or 48 or 49 or 53 or 54 or 55 or 56 or 57 or 58 or 59 or 60 or 61 or 62 or 63 or 64 or 65 or 66 or 67 or 68 or 69 or 70 or 71 or 72 or 73 or 74 or 75 or 76
84. 79 and 80 and 81 and 82 and 83
85. limit 84 to (human and english language)

**Global Health <1910 to 2021 Week 25>**

1. mental health.ti,ab,cc.
2. Mental Health/
3. exp Mental Disorders/
4. people with mental disabilities/
5. mental disorder*.ti,ab,cc.
6. mental* ill*.ti,ab,cc.
7. discrimination/
8. social discriminat*.ti,ab,cc.
9. prejudice.ti,ab,cc.
10. marginalization/
11. social marginali*.ti,ab,cc.
12. social isolation/
13. social stigma/
14. stigma*.ti,ab,cc.
15. stereotyp*.ti,ab,cc.
16. attitudes/
17. attitud*.ti,ab,cc.
18. social acceptance.ti,ab,cc.
19. social perception.ti,ab,cc.
20. social exclusion.ti,ab,cc.
21. engag*.ti,ab,cc.
22. (afghanistan or albania or algeria or american samoa or angola or "antigua and barbuda" or antigua or barbuda or argentina or armenia or armenian or aruba or azerbaijan or bahrain or bangladesh or barbados or republic of belarus or belarus or byelarus or belorussia or byelorussian or belize or british honduras or benin or dahomey or bhutan or bolivia or "bosnia and herzegovina" or bosnia or herzegovina or botswana or bechuanaland or brazil or brasil or bulgaria or burkina faso or burkina fasso or upper volta or burundi or urundi or cabo verde or cape verde or cambodia or kampuchea or khmer republic or cameroon or cameron or cameroun or central african republic or ubangi shari or chad or chile or china or colombia or comoros or comoro islands or iles comores or mayotte or democratic republic of the congo or democratic republic congo or congo or zaire or costa rica or "cote d’ivoire" or "cote d’ ivoire" or cote divoire or cote d ivoire or ivory coast or croatia or cuba or cyprus or czech republic or czechoslovakia or djibouti or french somaliland or dominica or dominican republic or ecuador or egypt or united arab republic or el salvador or equatorial guinea or spanish guinea or eritrea or estonia or eswatini or swaziland or ethiopia or fiji or gabon or gabonese republic or gambia or "georgia (republic)" or georgian or ghana or gold coast or gibraltar or greece or grenada or guam or guatemala or guinea or guinea bissau or guyana or british guiana or haiti or hispaniola or honduras or hungary or india or indonesia or timor or iran or iraq or isle of man or jamaica or jordan or kazakhstan or kazakh or kenya or "democratic people’s republic of korea" or republic of korea or north korea or south korea or korea or kosovo or kyrgyzstan or kirghizia or kirgizstan or kyrgyz republic or kirghiz or laos or lao pdr or "lao people's democratic republic" or latvia or lebanon or lebanese republic or lesotho or basutoland or liberia or libya or libyan arab jamahiriya or lithuania or macau or macao or republic of north macedonia or macedonia or madagascar or malagasy republic or malawi or nyasaland or malaysia or malay federation or malaya federation or maldives or indian ocean islands or indian ocean or mali or malta or micronesia or federated states of micronesia or kiribati or marshall islands or nauru or northern mariana islands or palau or tuvalu or mauritania or mauritius or mexico or moldova or moldovian or mongolia or montenegro or morocco or ifni or mozambique or portuguese east africa or myanmar or burma or namibia or nepal or netherlands antilles or nicaragua or niger or nigeria or oman or muscat or pakistan or panama or papua new guinea or new guinea or paraguay or peru or philippines or philipines or phillipines or phillippines or poland or "polish people's republic" or portugal or portuguese republic or puerto rico or romania or russia or russian federation or ussr or soviet union or union of soviet socialist republics or rwanda or ruanda or samoa or pacific islands or polynesia or samoan islands or navigator island or navigator islands or "sao tome and principe" or saudi arabia or senegal or serbia or seychelles or sierra leone or slovakia or slovak republic or slovenia or melanesia or solomon island or solomon islands or norfolk island or norfolk islands or somalia or south africa or south sudan or sri lanka or ceylon or "saint kitts and nevis" or "st. kitts and nevis" or saint lucia or "st. lucia" or "saint vincent and the grenadines" or saint vincent or "st. vincent" or grenadines or sudan or suriname or surinam or dutch guiana or netherlands guiana or syria or syrian arab republic or tajikistan or tadjikistan or tadzhikistan or tadzhik or tanzania or tanganyika or thailand or siam or timor leste or east timor or togo or togolese republic or tonga or "trinidad and tobago" or trinidad or tobago or tunisia or turkey or turkmenistan or turkmen or uganda or ukraine or uruguay or uzbekistan or uzbek or vanuatu or new hebrides or venezuela or vietnam or viet nam or middle east or west bank or gaza or palestine or yemen or yugoslavia or zambia or zimbabwe or northern rhodesia or global south or africa south of the sahara or sub-saharan africa or subsaharan africa or africa, central or central africa or africa, northern or north africa or northern africa or magreb or maghrib or sahara or africa, southern or southern africa or africa, eastern or east africa or eastern africa or africa, western or west africa or western africa or west indies or indian ocean islands or caribbean or central america or latin america or "south and central america" or south america or asia, central or central asia or asia, northern or north asia or northern asia or asia, southeastern or southeastern asia or south eastern asia or southeast asia or south east asia or asia, western or western asia or europe, eastern or east europe or eastern europe or developing country or developing countries or developing nation? or developing population? or developing world or less developed countr* or less developed nation? or less developed population? or less developed world or lesser developed countr* or lesser developed nation? or lesser developed population? or lesser developed world or under developed countr* or under developed nation? or under developed population? or under developed world or underdeveloped countr* or underdeveloped nation? or underdeveloped population? or underdeveloped world or middle income countr* or middle income nation? or middle income population? or low income countr* or low income nation? or low income population? or lower income countr* or lower income nation? or lower income population? or underserved countr* or underserved nation? or underserved population? or underserved world or under served countr* or under served nation? or under served population? or under served world or deprived countr* or deprived nation? or deprived population? or deprived world or poor countr* or poor nation? or poor population? or poor world or poorer countr* or poorer nation? or poorer population? or poorer world or developing econom* or less developed econom* or lesser developed econom* or under developed econom* or underdeveloped econom* or middle income econom* or low income econom* or lower income econom* or low gdp or low gnp or low gross domestic or low gross national or lower gdp or lower gnp or lower gross domestic or lower gross national or lmic or lmics or third world or lami countr* or transitional countr* or emerging economies or emerging nation?).ti,ab,cc,sh.
23. (afghan or afghans or afghani or albanian? or algerian? or american samoan? or angolan? or antiguan? or barbudan? or argentine? or argentinian? or argentinean? or armenian? or aruban? or azerbaijani? or bahraini? or bangladeshi? or bangalees or bajan? or belarusian? or byelorussian? or belizean? or beninese? or bhutanese or bolivian? or bosnian? or botswana or batswana or brazilian? or brasilian? or bulgarian? or burkinabe or burkinese or burundian? or cape verdean? or cabo verdean? or cambodian? or khmer or cameroonian? or central african? or chadian? or chilean? or chinese or colombian? or comorian? or congolese or costa rican? or ivorian? or croatian? or cuban? or cypriot? or czech? or djiboutian? or dominican? or ecuadorian? or egyptian? or salvadoran? or equatorial guinean? or equatoguinean? or eritrean? or estonian? or swazi? or swati? or ethiopian? or fijian or gabonese or gabonaise or gambian? or georgian? or ghanaian? or gibraltarian? or greek? or grenadian? or guamanian? or guatemalan? or guinean? or bissau guinean? or guyanese or haitian? or honduran? or hungarian? or indian? or indonesian? or iranian? or iraqian? or iraqi? or manx or jamaican? or jordanian? or kazakhstani? or kenyan? or kirabati or kirabatian? or north korean? or korean? or kosovar? or kosovan? or kyrgyz* or lao or laotian? or latvian? or lebanese or lesothan? or lesothonian? or mosotho or basotho or liberian? or libyan? or lithuanian? or macanese or macedonian? or malagasy or madagascan? or malawian? or malaysian? or maldivian? or malian? or maltese or marshallese? or mauritanian? or mauritian? or mexican? or micronesian? or moldovan? or mongolian? or mongol or montenegrin? or moroccan? or mozambican? or burmese or myanma or namibian? or nauruan? or nepali or nepalese or netherlands antillean? or nicaraguan? or nigerien? or nigerian? or northern mariana islander? or mariana? or omani? or pakistani? or palauan? or panamanian? or papua new guinean? or paraguayan? or peruvian? or philippine? or philipine? or phillipine? or phillippine? or filipino? or filipina? or polish or pole or poles or portuguese or puerto rican? or romanian? or russian? or soviet people or soviet population or rwandan? or rwandese or ruandan? or ruandese or samoan? or sao tomean? or santomean? or saudi arabian? or saudi? or senegalese or serbian? or montenegrin? or seychellois or seychelloise? or sierra leonean? or slovak? or slovene? or solomon islander? or somali? or south african? or south sudanese or sri lankan? or ceylonese or kittitian? or nevisian? or saint lucian? or vincentian? or sudanese or surinamese? or syrian? or tajik? or tajikistani? or tanzanian? or tanganyikan? or thai or timorese? or togolese or tongan? or trinidadian? or tobagonian? or tunisian? or turk? or turkish or turkmen? or tuvaluan? or ugandan? or ukrainian? or uruguayan? or uzbek? or vanuatu* or venezuelan? or vietnamese or yemeni? or yemenite? or yemenese or yugoslav? or yugoslavian? or zambian? or zimbabwean?).ti,ab,sh,cc.
24. community health services.sh.
25. program evaluation/
26. follow up/
27. health beliefs/
28. randomized controlled trials/
29. intervention/
30. health education/
31. clinical trial.ti,ab,cc.
32. (stigma* adj5 reduc*).ti,ab,cc.
33. (discrim* adj5 reduc*).ti,ab,cc.
34. campaigns/
35. intervent*.ti,ab,cc.
36. campaign*.ti,ab,cc.
37. ((improve* or change*) adj5 (knowledge* or attitude* or behavio*)).ti,ab,cc.
38. anti?stigma*.ti,ab,cc.
39. workshop*.ti,ab,cc.
40. workshops/
41. (community adj5 "mental health").ti,ab,cc.
42. pre-post.ti,ab,cc.
43. evaluat*.ti,ab,cc.
44. psychoeducat*.ti,ab,cc.
45. internet.sh.
46. social media/
47. videos/
48. mass media/
49. storytel*.ti,ab,cc.
50. blog*.ti,ab,cc.
51. social media.ti,ab,cc.
52. internet.ti,ab,cc.
53. presentation.ti,ab,cc.
54. indirect contact.ti,ab,cc.
55. social contact.ti,ab,cc.
56. extended contact.ti,ab,cc.
57. imagin* contact.ti,ab,cc.
58. vicario* contact.ti,ab,cc.
59. narrativ*.ti,ab,cc.
60. film*.ti,ab,cc.
61. video.ti,ab,cc.
62. theatr*.ti,ab,cc.
63. photo?voice.ti,ab,cc.
64. 1 or 2 or 3 or 4 or 5 or 6
65. 7 or 8 or 9 or 10 or 11 or 12 or 13 or 14 or 15 or 16 or 17 or 18 or 19 or 20 or 21
66. 22 or 23
67. 24 or 25 or 26 or 27 or 28 or 29 or 30 or 31 or 32 or 33 or 34 or 35 or 36 or 37 or 38 or 39 or 40 or 41 or 42 or 43 or 44
68. 21 or 24 or 34 or 36 or 39 or 40 or 41 or 45 or 46 or 47 or 48 or 49 or 50 or 51 or 52 or 53 or 54 or 55 or 56 or 57 or 58 or 59 or 60 or 61 or 62 or 63
69. 64 and 65 and 66 and 67 and 68
70. limit 69 to English language

**CINAHL 29.06.21**

| S76 | S71 AND S72 AND S73 AND S74 AND S75 | Limiters - English Language; Human Expanders - Apply equivalent subjects Search modes - Boolean/Phrase |
| --- | --- | --- |
| S75 | S23 OR S26 OR S39 OR S43 OR S44 OR S49 OR S50 OR S51 OR S52 OR S53 OR S54 OR S55 OR S56 OR S57 OR S58 OR S59 OR S60 OR S61 OR S62 OR S63 OR S64 OR S65 OR S66 OR S67 OR S68 OR S69 OR S70 | Search modes - Boolean/Phrase |
| S74 | S26 OR S27 OR S28 OR S29 OR S30 OR S31 OR S32 OR S33 OR S34 OR S35 OR S36 OR S37 OR S38 OR S39 OR S40 OR S41 OR S42 OR S43 OR S44 OR S45 OR S46 OR S47 | Search modes - Boolean/Phrase |
| S73 | S24 OR S25 | Search modes - Boolean/Phrase |
| S72 | S7 OR S8 OR S9 OR S10 OR S11 OR S12 OR S13 OR S14 OR S15 OR S16 OR S17 OR S18 OR S19 OR S20 OR S21 OR S22 OR S23 OR S48 | Search modes - Boolean/Phrase |
| S71 | S1 OR S2 OR S3 OR S4 OR S5 OR S6 | Search modes - Boolean/Phrase |
| S70 | photovoice | Search modes - Boolean/Phrase |
| S69 | theatr* | Search modes - Boolean/Phrase |
| S68 | video | Search modes - Boolean/Phrase |
| S67 | film* | Search modes - Boolean/Phrase |
| S66 | narrativ* | Search modes - Boolean/Phrase |
| S65 | vicario* contact | Search modes - Boolean/Phrase |
| S64 | imagin* contact | Search modes - Boolean/Phrase |
| S63 | extended contact | Search modes - Boolean/Phrase |
| S62 | social contact | Search modes - Boolean/Phrase |
| S61 | indirect contact | Search modes - Boolean/Phrase |
| S60 | presentation | Search modes - Boolean/Phrase |
| S59 | internet | Search modes - Boolean/Phrase |
| S58 | storytel* | Search modes - Boolean/Phrase |
| S57 | (MH "Communications Media") | Search modes - Boolean/Phrase |
| S56 | (MH "Multimedia") | Search modes - Boolean/Phrase |
| S55 | (MH "Audiorecording") | Search modes - Boolean/Phrase |
| S54 | (MH "Videorecording") | Search modes - Boolean/Phrase |
| S53 | (MH "Internet") | Search modes - Boolean/Phrase |
| S52 | (MH "Social Media") | Search modes - Boolean/Phrase |
| S51 | (MH "Storytelling") | Search modes - Boolean/Phrase |
| S50 | (MH "Narratives") | Search modes - Boolean/Phrase |
| S49 | (MH "Blogs") | Search modes - Boolean/Phrase |
| S48 | (MH "Attitude of Health Personnel") | Search modes - Boolean/Phrase |
| S47 | evaluat* | Search modes - Boolean/Phrase |
| S46 | pre-post | Search modes - Boolean/Phrase |
| S45 | change n5 stigma | Search modes - Boolean/Phrase |
| S44 | community n5 "mental health" | Search modes - Boolean/Phrase |
| S43 | workshop* | Search modes - Boolean/Phrase |
| S42 | anti-stigma | Search modes - Boolean/Phrase |
| S41 | (improv* or chang*) n5 (knowledge* or attitude* or behavio*) | Search modes - Boolean/Phrase |
| S40 | intervent* | Search modes - Boolean/Phrase |
| S39 | campaign* | Search modes - Boolean/Phrase |
| S38 | discrim* n5 reduc* | Search modes - Boolean/Phrase |
| S37 | stigma* n5 reduc* | Search modes - Boolean/Phrase |
| S36 | clinical trial | Search modes - Boolean/Phrase |
| S35 | psychoeducation | Search modes - Boolean/Phrase |
| S34 | (MH "Psychoeducation") | Search modes - Boolean/Phrase |
| S33 | (MH "Health Education") | Search modes - Boolean/Phrase |
| S32 | (MH "Internet-Based Intervention") | Search modes - Boolean/Phrase |
| S31 | (MH "Randomized Controlled Trials") | Search modes - Boolean/Phrase |
| S30 | (MH "Health Knowledge") | Search modes - Boolean/Phrase |
| S29 | (MH "Program Evaluation") | Search modes - Boolean/Phrase |
| S28 | (MH "Prospective Studies") | Search modes - Boolean/Phrase |
| S27 | (MH "Evaluation Research") | Search modes - Boolean/Phrase |
| S26 | (MH "Community Mental Health Services") | Search modes - Boolean/Phrase |
| S25 | afghan or afghans or afghani or albanian? algerian? or american samoan? or angolan? or antiguan? or barbudan? or argentine? or argentinian? or argentinean? or armenian? or aruban? or azerbaijani? or bahraini? or bangladeshi? or bangalees or bajan? or belarusian? or byelorussian? or belizean? or beninese? or bhutanese or bolivian? or bosnian? or botswana or batswana or brazilian? or brasilian? or bulgarian? or burkinabe or burkinese or burundian? or cape verdean? or cabo verdean? or cambodian? or khmer or cameroonian? or central african? or chadian? or chilean? or chinese or colombian? or comorian? or congolese or costa rican? or ivorian? or croatian? or cuban? or cypriot? or czech? or djiboutian? or dominican? or ecuadorian? or egyptian? or salvadoran? or equatorial guinean? or equatoguinean? or eritrean? or estonian? or swazi? or swati? or ethiopian? or fijian or gabonese or gabonaise or gambian? or georgian? or ghanaian? or gibraltarian? or greek? or grenadian? or guamanian? or guatemalan? or guinean? or bissau guinean? or guyanese or haitian? or honduran? or hungarian? or indian? or indonesian? or iranian? or iraqian? or iraqi? or manx or jamaican? or jordanian? or kazakhstani? or kenyan? or kirabati or kirabatian? or north korean? or korean? or kosovar? or kosovan? or kyrgyz* or lao or laotian? or latvian? or lebanese or lesothan? or lesothonian? or mosotho or basotho or liberian? or libyan? or lithuanian? or macanese or macedonian? or malagasy or madagascan? or malawian? or malaysian? or maldivian? or malian? or maltese or marshallese? or mauritanian? or mauritian? or mexican? or micronesian? or moldovan? or mongolian? or mongol or montenegrin? or moroccan? or mozambican? or burmese or myanma or namibian? or nauruan? or nepali or nepalese or netherlands antillean? or nicaraguan? or nigerien? or nigerian? or northern mariana islander? or mariana? or omani? or pakistani? or palauan? or panamanian? or papua new guinean? or paraguayan? or peruvian? or philippine? or philipine? or phillipine? or phillippine? or filipino? or filipina? or polish or pole or poles or portuguese or puerto rican? or romanian? or russian? or soviet people or soviet population or rwandan? or rwandese or ruandan? or ruandese or samoan? or sao tomean? or santomean? or saudi arabian? or saudi? or senegalese or serbian? or montenegrin? or seychellois or seychelloise? or sierra leonean? or slovak? or slovene? or solomon islander? or somali? or south african? or south sudanese or sri lankan? or ceylonese or kittitian? or nevisian? or saint lucian? or vincentian? or sudanese or surinamese? or syrian? or tajik? or tajikistani? or tanzanian? or tanganyikan? or thai or timorese? or togolese or tongan? or trinidadian? or tobagonian? or tunisian? or turk? or turkish or turkmen? or tuvaluan? or ugandan? or ukrainian? or uruguayan? or uzbek? or vanuatu* or venezuelan? or vietnamese or yemeni? or yemenite? or yemenese or yugoslav? or yugoslavian? or zambian? or zimbabwean? | Expanders - Apply equivalent subjects Search modes - Boolean/Phrase |
| S24 | afghanistan or albania or algeria or american samoa or angola or "antigua and barbuda" or antigua or barbuda or argentina or armenia or armenian or aruba or azerbaijan or bahrain or bangladesh or barbados or republic of belarus or belarus or byelarus or belorussia or byelorussian or belize or british honduras or benin or dahomey or bhutan or bolivia or "bosnia and herzegovina" or bosnia or herzegovina or botswana or bechuanaland or brazil or brasil or bulgaria or burkina faso or burkina fasso or upper volta or burundi or urundi or cabo verde or cape verde or cambodia or kampuchea or khmer republic or cameroon or cameron or cameroun or central african republic or ubangi shari or chad or chile or china or colombia or comoros or comoro islands or iles comores or mayotte or democratic republic of the congo or democratic republic congo or congo or zaire or costa rica or "cote d’ivoire" or "cote d’ ivoire" or cote divoire or cote d ivoire or ivory coast or croatia or cuba or cyprus or czech republic or czechoslovakia or djibouti or french somaliland or dominica or dominican republic or ecuador or egypt or united arab republic or el salvador or equatorial guinea or spanish guinea or eritrea or estonia or eswatini or swaziland or ethiopia or fiji or gabon or gabonese republic or gambia or "georgia (republic)" or georgian or ghana or gold coast or gibraltar or greece or grenada or guam or guatemala or guinea or guinea bissau or guyana or british guiana or haiti or hispaniola or honduras or hungary or india or indonesia or timor or iran or iraq or isle of man or jamaica or jordan or kazakhstan or kazakh or kenya or "democratic people’s republic of korea" or republic of korea or north korea or south korea or korea or kosovo or kyrgyzstan or kirghizia or kirgizstan or kyrgyz republic or kirghiz or laos or lao pdr or "lao people's democratic republic" or latvia or lebanon or lebanese republic or lesotho or basutoland or liberia or libya or libyan arab jamahiriya or lithuania or macau or macao or republic of north macedonia or macedonia or madagascar or malagasy republic or malawi or nyasaland or malaysia or malay federation or malaya federation or maldives or indian ocean islands or indian ocean or mali or malta or micronesia or federated states of micronesia or kiribati or marshall islands or nauru or northern mariana islands or palau or tuvalu or mauritania or mauritius or mexico or moldova or moldovian or mongolia or montenegro or morocco or ifni or mozambique or portuguese east africa or myanmar or burma or namibia or nepal or netherlands antilles or nicaragua or niger or nigeria or oman or muscat or pakistan or panama or papua new guinea or new guinea or paraguay or peru or philippines or philipines or phillipines or phillippines or poland or "polish people's republic" or portugal or portuguese republic or puerto rico or romania or russia or russian federation or ussr or soviet union or union of soviet socialist republics or rwanda or ruanda or samoa or pacific islands or polynesia or samoan islands or navigator island or navigator islands or "sao tome and principe" or saudi arabia or senegal or serbia or seychelles or sierra leone or slovakia or slovak republic or slovenia or melanesia or solomon island or solomon islands or norfolk island or norfolk islands or somalia or south africa or south sudan or sri lanka or ceylon or "saint kitts and nevis" or "st. kitts and nevis" or saint lucia or "st. lucia" or "saint vincent and the grenadines" or saint vincent or "st. vincent" or grenadines or sudan or suriname or surinam or dutch guiana or netherlands guiana or syria or syrian arab republic or tajikistan or tadjikistan or tadzhikistan or tadzhik or tanzania or tanganyika or thailand or siam or timor leste or east timor or togo or togolese republic or tonga or "trinidad and tobago" or trinidad or tobago or tunisia or turkey or turkmenistan or turkmen or uganda or ukraine or uruguay or uzbekistan or uzbek or vanuatu or new hebrides or venezuela or vietnam or viet nam or middle east or west bank or gaza or palestine or yemen or yugoslavia or zambia or zimbabwe or northern rhodesia or global south or africa south of the sahara or sub-saharan africa or subsaharan africa or africa, central or central africa or africa, northern or north africa or northern africa or magreb or maghrib or sahara or africa, southern or southern africa or africa, eastern or east africa or eastern africa or africa, western or west africa or western africa or west indies or indian ocean islands or caribbean or central america or latin america or "south and central america" or south america or asia, central or central asia or asia, northern or north asia or northern asia or asia, southeastern or southeastern asia or south eastern asia or southeast asia or south east asia or asia, western or western asia or europe, eastern or east europe or eastern europe or developing country or developing countries or developing nation? or developing population? or developing world or less developed countr* or less developed nation? or less developed population? or less developed world or lesser developed countr* or lesser developed nation? or lesser developed population? or lesser developed world or under developed countr* or under developed nation? or under developed population? or under developed world or underdeveloped countr* or underdeveloped nation? or underdeveloped population? or underdeveloped world or middle income countr* or middle income nation? or middle income population? or low income countr* or low income nation? or low income population? or lower income countr* or lower income nation? or lower income population? or underserved countr* or underserved nation? or underserved population? or underserved world or under served countr* or under served nation? or under served population? or under served world or deprived countr* or deprived nation? or deprived population? or deprived world or poor countr* or poor nation? or poor population? or poor world or poorer countr* or poorer nation? or poorer population? or poorer world or developing econom* or less developed econom* or lesser developed econom* or under developed econom* or underdeveloped econom* or middle income econom* or low income econom* or lower income econom* or low gdp or low gnp or low gross domestic or low gross national or lower gdp or lower gnp or lower gross domestic or lower gross national or lmic or lmics or third world or lami countr* or transitional countr* or emerging economies or emerging nation? | Expanders - Apply equivalent subjects Search modes - Boolean/Phrase |
| S23 | engag* | Search modes - Boolean/Phrase |
| S22 | social exclusion | Search modes - Boolean/Phrase |
| S21 | social perception | Search modes - Boolean/Phrase |
| S20 | social acceptance | Search modes - Boolean/Phrase |
| S19 | attitud* | Search modes - Boolean/Phrase |
| S18 | (MH "Attitude to Mental Illness") | Search modes - Boolean/Phrase |
| S17 | (MH "Attitude") | Search modes - Boolean/Phrase |
| S16 | stereotyp* | Search modes - Boolean/Phrase |
| S15 | (MH "Stereotyping") | Search modes - Boolean/Phrase |
| S14 | stigma* | Search modes - Boolean/Phrase |
| S13 | (MH "Stigma") | Search modes - Boolean/Phrase |
| S12 | (MH "Social Isolation") | Search modes - Boolean/Phrase |
| S11 | social discrimination | Search modes - Boolean/Phrase |
| S10 | (MH "Discrimination") | Search modes - Boolean/Phrase |
| S9 | social marginalization | Search modes - Boolean/Phrase |
| S8 | prejudice | Search modes - Boolean/Phrase |
| S7 | (MH "Prejudice") | Search modes - Boolean/Phrase |
| S6 | mental* ill* | Search modes - Boolean/Phrase |
| S5 | (MH "Mentally Disabled Persons") | Search modes - Boolean/Phrase |
| S4 | mental disorder* | Search modes - Boolean/Phrase |
| S3 | (MH "Mental Disorders") | Search modes - Boolean/Phrase |
| S2 | mental health | Search modes - Boolean/Phrase |
| S1 | (MH "Mental Health") | Search modes - Boolean/Phrase |

**CENTRAL Search: 29/06/2021**

#1 MeSH descriptor: [Mental Health] this term only

#2 (mental health):ti,ab,kw

#3 MeSH descriptor: [Mental Disorders] this term only

#4 (mental disorder*):ti,ab,kw

#5 MeSH descriptor: [Mentally Ill Persons] this term only

#6 (mental* ill*):ti,ab,kw

#7 MeSH descriptor: [Prejudice] this term only

#8 (prejudice):ti,ab,kw

#9 MeSH descriptor: [Discrimination, Psychological] explode all trees

#10 (social discriminat*):ti,ab,kw

#11 MeSH descriptor: [Social Marginalization] this term only

#12 (social marginali*):ti,ab,kw

#13 MeSH descriptor: [Social Isolation] this term only

#14 MeSH descriptor: [Social Stigma] this term only

#15 (stigma*):ti,ab,kw

#16 MeSH descriptor: [Stereotyping] this term only

#17 (stereotyp*):ti,ab,kw

#18 MeSH descriptor: [Attitude] this term only

#19 (attitud*):ti,ab,kw

#20 MeSH descriptor: [Attitude of Health Personnel] this term only

#21 (social acceptance):ti,ab,kw

#22 (social perception):ti,ab,kw

#23 (social exclusion):ti,ab,kw

#24 (afghanistan OR albania OR algeria OR "american samoa" OR angola OR "antigua and barbuda" OR antigua OR barbuda OR argentina OR armenia OR armenian OR aruba OR azerbaijan OR bahrain OR bangladesh OR barbados OR "republic of belarus" OR belarus OR byelarus OR belorussia OR byelorussian OR belize OR "british honduras" OR benin OR dahomey OR bhutan OR bolivia OR "bosnia and herzegovina" OR bosnia OR herzegovina OR botswana OR bechuanaland OR brazil OR brasil OR bulgaria OR "burkina faso" OR "burkina fasso" OR "upper volta" OR burundi OR urundi OR "cabo verde" OR "cape verde" OR cambodia OR kampuchea OR "khmer republic" OR cameroon OR cameron OR cameroun OR "central african republic" OR "ubangi shari" OR chad OR chile OR china OR colombia OR comoros OR "comoro islands" OR "iles comores" OR mayotte OR "democratic republic of the congo" OR "democratic republic congo" OR congo OR zaire OR "costa rica" OR "cote d’ivoire" OR "cote d’ ivoire" OR "cote divoire" OR "cote d ivoire" OR "ivory coast" OR croatia OR cuba OR cyprus OR "czech republic" OR czechoslovakia OR djibouti OR "french somaliland" OR dominica OR "dominican republic" OR ecuador OR egypt OR "united arab republic" OR "el salvador" OR "equatorial guinea" OR "spanish guinea" OR eritrea OR estonia OR eswatini OR swaziland OR ethiopia OR fiji OR gabon OR "gabonese republic" OR gambia OR "georgia (republic)" OR georgia OR georgian OR ghana OR "gold coast" OR gibraltar OR greece OR grenada OR guam OR guatemala OR guinea OR "guinea bissau" OR guyana OR "british guiana" OR haiti OR hispaniola OR honduras OR hungary OR india OR indonesia OR timor OR iran OR iraq OR "isle of man" OR jamaica OR jordan OR kazakhstan OR kazakh OR kenya OR "democratic people’s republic of korea" OR "republic of korea" OR north korea OR south korea OR korea OR kosovo OR kyrgyzstan OR kirghizia OR kirgizstan OR "kyrgyz republic" OR kirghiz OR laos OR "lao pdr" OR "lao people's democratic republic" OR latvia OR lebanon OR "lebanese republic" OR lesotho OR basutoland OR liberia OR libya OR "libyan arab jamahiriya" OR lithuania OR macau OR macao OR "republic of north macedonia" OR macedonia OR madagascar OR "malagasy republic" OR malawi OR nyasaland OR malaysia OR "malay federation" OR "malaya federation" OR maldives OR "indian ocean islands" OR "indian ocean" OR mali OR malta OR micronesia OR "federated states of micronesia" OR kiribati OR "marshall islands" OR nauru OR "northern mariana islands" OR palau OR tuvalu OR mauritania OR mauritius OR mexico OR moldova OR moldovian OR mongolia OR montenegro OR morocco OR ifni OR mozambique OR "portuguese east africa" OR myanmar OR burma OR namibia OR nepal OR "netherlands antilles" OR nicaragua OR niger OR nigeria OR oman OR muscat OR pakistan OR panama OR "papua new guinea" OR paraguay OR peru OR philippines OR philipines OR phillipines OR phillippines OR poland OR "polish people's republic" OR portugal OR "portuguese republic" OR "puerto rico" OR romania OR russia OR "russian federation" OR ussr OR "soviet union" OR "union of soviet socialist republics" OR rwanda OR ruanda OR samoa OR "pacific islands" OR polynesia OR "samoan islands" OR "navigator island" OR "navigator islands" OR "sao tome and principe" OR "saudi arabia" OR senegal OR serbia OR seychelles OR "sierra leone" OR slovakia OR "slovak republic" OR slovenia OR melanesia OR "solomon island" OR "solomon islands" OR "norfolk island" OR "norfolk islands" OR somalia OR "south africa" OR "south sudan" OR "sri lanka" OR ceylon OR "saint kitts and nevis" OR "st. kitts and nevis" OR "saint lucia" OR "st. lucia" OR "saint vincent and the grenadines" OR "saint vincent" OR "st. vincent" OR grenadines OR sudan OR suriname OR surinam OR "dutch guiana" OR "netherlands guiana" OR syria OR "syrian arab republic" OR tajikistan OR tadjikistan OR tadzhikistan OR tadzhik OR tanzania OR tanganyika OR thailand OR siam OR "timor leste" OR "east timor" OR togo OR "togolese republic" OR tonga OR "trinidad and tobago" OR trinidad OR tobago OR tunisia OR turkey OR turkmenistan OR turkmen OR uganda OR ukraine OR uruguay OR uzbekistan OR uzbek OR vanuatu OR "new hebrides" OR venezuela OR vietnam OR "viet nam" OR "middle east" OR "west bank" OR gaza OR palestine OR yemen OR yugoslavia OR zambia OR zimbabwe OR "northern rhodesia" OR "global south" OR "africa south of the sahara" OR "sub saharan africa" OR "subsaharan africa" OR "africa, central" OR "central africa" OR "africa, northern" OR "north africa" OR "northern africa" OR magreb OR maghrib OR sahara OR "africa, southern" OR "southern africa" OR "africa, eastern" OR "east africa" OR "eastern africa" OR "africa, western" OR "west africa" OR "western africa" OR "west indies" OR "indian ocean islands" OR caribbean OR "central america" OR "latin america" OR "south and central america" OR "south america" OR "asia, central" OR "central asia" OR "asia, northern" OR "north asia" OR "northern asia" OR "asia, southeastern" OR "southeastern asia" OR "south eastern asia" OR "southeast asia" OR "south east asia" OR "asia, western" OR "western asia" OR "europe, eastern" OR "east europe" OR "eastern europe" OR "developing country" OR "developing countries" OR "developing nation" OR "developing nations" OR "developing population" OR "developing populations" OR "developing world" OR "less developed country" OR "less developed countries" OR "less developed nation" OR "less developed nations" OR "less developed population" OR "less developed populations" OR "less developed world" OR "lesser developed country" OR "lesser developed countries" OR "lesser developed nation" OR "lesser developed nations" OR "lesser developed population" OR "lesser developed populations" OR "lesser developed world" OR "under developed country" OR "under developed countries" OR "under developed nation" OR "under developed nations" OR "under developed population" OR "under developed populations" OR "under developed world" OR "underdeveloped country" OR "underdeveloped countries" OR "underdeveloped nation" OR "underdeveloped nations" OR "underdeveloped population" OR "underdeveloped populations" OR "underdeveloped world" OR "middle income country" OR "middle income countries" OR "middle income nation" OR "middle income nations" OR "middle income population" OR "middle income populations" OR "low income country" OR "low income countries" OR "low income nation" OR "low income nations" OR "low income population" OR "low income populations" OR "lower income country" OR "lower income countries" OR "lower income nation" OR "lower income nations" OR "lower income population" OR "lower income populations" OR "underserved country" OR "underserved countries" OR "underserved nation" OR "underserved nations" OR "underserved population" OR "underserved populations" OR "underserved world" OR "under served country" OR "under served countries" OR "under served nation" OR "under served nations" OR "under served population" OR "under served populations" OR "under served world" OR "deprived country" OR "deprived countries" OR "deprived nation" OR "deprived nations" OR "deprived population" OR "deprived populations" OR "deprived world" OR "poor country" OR "poor countries" OR "poor nation" OR "poor nations" OR "poor population" OR "poor populations" OR "poor world" OR "poorer country" OR "poorer countries" OR "poorer nation" OR "poorer nations" OR "poorer population" OR "poorer populations" OR "poorer world" OR "developing economy" OR "developing economies" OR "less developed economy" OR "less developed economies" OR "lesser developed economy" OR "lesser developed economies" OR "under developed economy" OR "under developed economies" OR "underdeveloped economy" OR "underdeveloped economies" OR "middle income economy" OR "middle income economies" OR "low income economy" OR "low income economies" OR "lower income economy" OR "lower income economies" OR "low gdp" OR "low gnp" OR "low gross domestic" OR "low gross national" OR "lower gdp" OR "lower gnp" OR "lower gross domestic" OR "lower gross national" OR lmic OR lmics OR "third world" OR "lami country" OR "lami countries" OR "transitional country" OR "transitional countries" OR "emerging economies" OR "emerging nation" OR "emerging nations"):ti,ab,kw

#25 (afghan OR afghans OR afghani OR albanian OR albanians OR algerian OR algerians OR "american samoan" OR "american samoans" OR angolan OR angolans OR antiguan OR antiguans OR barbudan OR berbudans OR argentine OR argentines OR argentinian OR argentinians OR argentinean OR argentineans OR armenian OR armenians OR aruban OR arubans OR azerbaijani OR azerbaijanis OR bahraini OR bahrainis OR bangladeshi OR bangladeshis OR bangalees OR bajan OR bajans OR belarusian OR belarusians OR byelorussian OR byelorussians OR belizean OR belizeans OR beninese OR benineses OR bhutanese OR bolivian OR bolivians OR bosnian OR bosnians OR botswana OR batswana OR brazilian OR brazilians OR brasilian OR brasilians OR bulgarian OR bulgarians OR burkinabe OR burkinese OR burundian OR burundians OR "cape verdean" OR "cape verdeans" OR "cabo verdean" OR "cabo verdeans" OR cambodian OR cambodians OR khmer OR cameroonian OR cameroonians OR "central african" OR "central africans" OR chadian OR chadians OR chilean OR chileans OR chinese OR colombian OR colombians OR comorian OR comorians OR congolese OR "costa rican" OR "costa ricans" OR ivorian OR ivorians OR croatian OR croatians OR cuban OR cubans OR cypriot OR cypriots OR czech OR czechs OR djiboutian OR djiboutians OR dominican OR dominicans OR ecuadorian OR ecuadorians OR egyptian OR egyptians OR salvadoran OR salvadorans OR "equatorial guinean" OR "equatorial guineans" OR equatoguinean OR equatoguineans OR eritrean OR eritreans OR estonian OR estonians OR swazi OR swazis OR swati OR swatis OR ethiopian OR ethiopians OR fijian OR fijians OR gabonese OR gabonaise OR gambian OR gambians OR georgian OR georgians OR ghanaian OR ghanaians OR gibraltarian OR gibraltarians OR greek OR greeks OR grenadian OR grenadians OR guamanian OR guamanians OR guatemalan OR guatemalans OR guinean OR guineans OR "bissau guinean" OR "bissau guineans" OR guyanese OR haitian OR haitians OR honduran OR hondurans OR hungarian OR hungarians OR indian OR indians OR indonesian OR indonesians OR iranian OR iranians OR iraqian OR iraqians OR iraqi OR iraqis OR manx OR jamaican OR jamaicans OR jordanian OR jordanians OR kazakhstani OR kazakhstanis OR kenyan OR kenyans OR kirabati OR kirabatian OR kirabatians OR "north korean" OR "north koreans" OR korean OR koreans OR kosovar OR kosovars OR kosovan OR kosovans OR kyrgyzstani OR kyrgyzstanis OR kyrgyz OR lao OR laotian OR laotians OR latvian OR latvians OR lebanese OR lesothan OR lesothans OR lesothonian OR lesothonians OR mosotho OR basotho OR liberian OR liberians OR libyan OR libyans OR lithuanian OR lithuanians OR macanese OR macedonian OR macedonians OR malagasy OR madagascan OR madagascans OR malawian OR malawians OR malaysian OR malaysians OR maldivian OR maldivians OR malian OR malians OR maltese OR marshallese OR marshalleses OR mauritanian OR mauritanians OR mauritian OR mauritians OR mexican OR mexicans OR micronesian OR micronesians OR moldovan OR moldovans OR mongolian OR mongolians OR mongol OR montenegrin OR montenegrins OR moroccan OR moroccans OR mozambican OR mozambicans OR burmese OR myanma OR namibian OR namibians OR nauruan OR nauruans OR nepali OR nepalese OR "netherlands antillean" OR "netherlands antilleans" OR nicaraguan OR nicaraguans OR nigerien OR nigeriens OR nigerian OR nigerians OR "northern mariana islander" OR "northern mariana islanders" OR mariana OR marianas OR omani OR omanis OR pakistani OR pakistanis OR palauan OR palauans OR panamanian OR panamanians OR "papua new guinean" OR "papua new guineans" OR paraguayan OR paraguayans OR peruvian OR peruvians OR philippine OR philippines OR philipine OR philipines OR phillipine OR phillipines OR phillippine OR phillippines OR filipino OR filipinos OR filipina OR filipinas OR polish OR pole OR poles OR portuguese OR "puerto rican" OR "puerto ricans" OR romanian OR romanians OR russian OR russians OR "soviet people" OR "soviet population" OR rwandan OR rwandans OR rwandese OR ruandan OR ruandans OR ruandese OR samoan OR samoans OR "sao tomean" OR "sao tomeans" OR santomean OR santomeans OR "saudi arabian" OR "saudi arabians" OR saudi OR saudis OR senegalese OR serbian OR serbians OR montenegrin OR montenegrins OR seychellois OR seychelloise OR seychelloises OR "sierra leonean" OR "sierra leoneans" OR slovak OR slovaks OR slovene OR slovenes OR "solomon islander" OR "solomon islanders" OR somali OR somalis OR "south african" OR "south africans" OR "south sudanese" OR "sri lankan" OR "sri lankans" OR ceylonese OR kittitian OR kittitians OR nevisian OR nevisians OR "saint lucian" OR "saint lucians" OR vincentian OR vincentians OR sudanese OR surinamese OR surinameses OR syrian OR syrians OR tajik OR tajiks OR tajikistani OR tajikistanis OR tanzanian OR tanzanians OR tanganyikan OR tanganyikans OR thai OR timorese OR timoreses OR togolese OR tongan OR tongans OR trinidadian OR trinidadians OR tobagonian OR tobagonians OR tunisian OR tunisians OR turk OR turks OR turkish OR turkmen OR turkmens OR tuvaluan OR tuvaluans OR ugandan OR ugandans OR ukrainian OR ukrainians uruguayan OR uruguayans OR uzbek OR uzbeks OR vanuatu OR vanuatuan OR vanuatuans OR venezuelan OR venezuelans OR vietnamese OR yemeni OR yemenis OR yemenite OR yemenites OR yemenese OR yugoslav OR yugoslavs OR yugoslavian OR yugoslavians OR zambian OR zambians OR zimbabwean OR zimbabweans):ti,ab,kw

#26 MeSH descriptor: [Community Mental Health Services] this term only

#27 MeSH descriptor: [Evaluation Study] this term only

#28 MeSH descriptor: [Follow-Up Studies] this term only

#29 MeSH descriptor: [Program Evaluation] this term only

#30 MeSH descriptor: [Health Knowledge, Attitudes, Practice] this term only

#31 MeSH descriptor: [Randomized Controlled Trial] this term only

#32 MeSH descriptor: [Internet-Based Intervention] this term only

#33 MeSH descriptor: [Health Education] explode all trees

#34 (clinical trial):ti,ab,kw

#35 ((stigma* near/5 reduc*)):ti,ab,kw

#36 ((discrim* near/5 reduc*)):ti,ab,kw

#37 (campaign*):ti,ab,kw

#38 (intervent*):ti,ab,kw

#39 (psychoeducation):ti,ab,kw

#40 (((improve* or change*) near/5 (knowledge* or attitude* or behavio*))):ti,ab,kw

#41 (anti?stigma*):ti,ab,kw

#42 (workshop*):ti,ab,kw

#43 ((community near/5 "mental health")):ti,ab,kw

#44 ((change near/5 stigma)):ti,ab,kw

#45 (pre-post):ti,ab,kw

#46 (evaluat*):ti,ab,kw

#47 MeSH descriptor: [Blogging] explode all trees

#48 MeSH descriptor: [Narration] this term only

#49 MeSH descriptor: [Personal Narrative] this term only

#50 MeSH descriptor: [Social Media] this term only

#51 MeSH descriptor: [Internet] this term only

#52 MeSH descriptor: [Multimedia] this term only

#53 (storytel*):ti,ab,kw

#54 (blog*):ti,ab,kw

#55 (social media):ti,ab,kw

#56 (internet):ti,ab,kw

#57 (presentation):ti,ab,kw

#58 (indirect contact):ti,ab,kw

#59 (social contact):ti,ab,kw

#60 (extended contact):ti,ab,kw

#61 (imagin* contact):ti,ab,kw

#62 (vicario* contact):ti,ab,kw

#63 (narrativ*):ti,ab,kw

#64 (film*):ti,ab,kw

#65 (video):ti,ab,kw

#66 (theatr*):ti,ab,kw

#67 (photo?voice):ti,ab,kw

#68 (engag*):ti,ab,kw

#69 #1 or #2 or #3 or #4 or #5 or #6

#70 #7 or #8 or #9 or #10 or #11 or #12 or #13 or #14 or #15 or #16 or #17 or #18 or #19 or #20 or #21 or #22 or #23 or #68

#71 #24 or #25

#72 #26 or #27 or #28 or #29 or #30 or #31 or #32 or #33 or #34 or #35 or #36 or #37 or #38 or #39 or #40 or #41 or #42 or #43 or #44 or #45 or #46

#73 #26 or #37 or #42 or #43 or #47 or #48 or #49 or #50 or #51 or #52 or #53 or #54 or #55 or #56 or #57 or #58 or #59 or #60 or #61 or #62 or #63 or #64 or #65 or #66 or #67 or #68

#74 #69 and #70 and #71 and #72 and #7

### List of Stigma-Related Reviews used for citation chaining

1. Clay, J., Eaton, J., Gronholm, P. C., Semrau, M., & Votruba, N. (2020). Core components of mental health stigma reduction interventions in low-and middle-income countries: a systematic review. *Epidemiology and psychiatric sciences*, *29*
2. Rao, D., Elshafei, A., Nguyen, M., Hatzenbuehler, M. L., Frey, S., & Go, V. F. (2019). A systematic review of multi-level stigma interventions: state of the science and future directions. *BMC medicine*, *17*(1), 1-11.
3. Ando, S., Clement, S., Barley, E. A., & Thornicroft, G. (2011). The simulation of hallucinations to reduce the stigma of schizophrenia: A systematic review. *Schizophrenia Research*, *133*(1-3), 8-16.
4. Janoušková, M., Tušková, E., Weissová, A., Trančík, P., Pasz, J., Evans-Lacko, S., & Winkler, P. (2017). Can video interventions be used to effectively destigmatize mental illness among young people? A systematic review. *European Psychiatry*, *41*(1), 1-9.
5. Büchter, R. B., & Messer, M. (2017). Interventions for reducing self-stigma in people with mental illnesses: a systematic review of randomized controlled trials. *GMS German Medical Science*, *15*.
6. Clement, S., Lassman, F., Barley, E., Evans‐Lacko, S., Williams, P., Yamaguchi, S., Slade, M., Rusch, N., & Thornicroft, G. (2013). Mass media interventions for reducing mental health‐related stigma. *Cochrane Database of Systematic Reviews*, (7).
7. Corrigan, P. W., Morris, S. B., Michaels, P. J., Rafacz, J. D., & Rüsch, N. (2012). Challenging the public stigma of mental illness: a meta-analysis of outcome studies. *Psychiatric services*, *63*(10), 963-973.
8. Morgan, A. J., Reavley, N. J., Ross, A., San Too, L., & Jorm, A. F. (2018). Interventions to reduce stigma towards people with severe mental illness: Systematic review and meta-analysis. *Journal of psychiatric research*, *103*, 120-133.
9. Morgan, A. J., Ross, A., & Reavley, N. J. (2018). Systematic review and meta-analysis of Mental Health First Aid training: Effects on knowledge, stigma, and helping behaviour. *PloS one*, *13*(5), e0197102.
10. Thornicroft, G., Mehta, N., Clement, S., Evans-Lacko, S., Doherty, M., Rose, D., Koschorke, M., Shidhaye, R., O’Reilly, C., & Henderson, C. (2016). Evidence for effective interventions to reduce mental-health-related stigma and discrimination. *The Lancet*, *387*(10023), 1123-1132.
11. Xu, Z., Rüsch, N., Huang, F., & Koesters, M. (2017). Challenging mental health related stigma in China: systematic review and meta-analysis. I. Interventions among the general public. *Psychiatry research*, *255*, 449-456.
12. Xu, Z., Huang, F., Koesters, M., & Ruesch, N. (2017). Challenging mental health related stigma in China: Systematic review and meta-analysis. II. Interventions among people with mental illness. *Psychiatry Research*, *255*, 457-464.
13. Seroalo, K. B., Du Plessis, E., Koen, M. P., & Koen, V. (2014). A critical synthesis of interventions to reduce stigma attached to mental illness. *Health SA Gesondheid*, *19*(1), 1-10.
14. Mehta, N., Clement, S., Marcus, E., Stona, A. C., Bezborodovs, N., Evans-Lacko, S., Palacious, J.,Docherty, M., Barley, E., Rose, D., & Thornicroft, G. (2015). Evidence for effective interventions to reduce mental health-related stigma and discrimination in the medium and long term: systematic review. *The British Journal of Psychiatry*, *207*(5), 377-384.
15. Gronholm, P. C., Henderson, C., Deb, T., & Thornicroft, G. (2017). Interventions to reduce discrimination and stigma: the state of the art. *Social psychiatry and psychiatric epidemiology*, *52*(3), 249-258.
16. Clement, S., Lassman, F., Barley, E., Evans‐Lacko, S., Williams, P., Yamaguchi, S., Slade, M., Rusch, N., & Thornicroft, G. (2013). Mass media interventions for reducing mental health‐related stigma. *Cochrane Database of Systematic Reviews*, (7).
17. Mansouri, N., Gharaee, B., Shariat, S. V., Bolhari, J., Nooraie, R. Y., Rahimi-Movaghar, A., & Alirezaie, N. (2009). The change in attitude and knowledge of health care personnel and general population following trainings provided during integration of mental health in Primary Health Care in Iran: a systematic review. *International Journal of Mental Health Systems*, *3*(1), 1-7.
18. Kemp, C. G., Jarrett, B. A., Kwon, C. S., Song, L., Jetté, N., Sapag, J. C., Bass, J., Murray, L., Rao, D., & Baral, S. (2019). Implementation science and stigma reduction interventions in low-and middle-income countries: a systematic review. *BMC medicine*, *17*(1), 1-18.
19. Heim, E., Kohrt, B. A., Koschorke, M., Milenova, M., & Thornicroft, G. (2020). Reducing mental health-related stigma in primary health care settings in low-and middle-income countries: a systematic review. *Epidemiology and psychiatric sciences*, *29*.
20. Heim, E., Henderson, C., Kohrt, B. A., Koschorke, M., Milenova, M., & Thornicroft, G. (2020). Reducing mental health-related stigma among medical and nursing students in low-and middle-income countries: a systematic review. *Epidemiology and psychiatric sciences*, *29*.
21. Griffiths, K. M., Carron‐Arthur, B., Parsons, A., & Reid, R. (2014). Effectiveness of programs for reducing the stigma associated with mental disorders. A meta‐analysis of randomized controlled trials. *World psychiatry*, *13*(2), 161-175.
22. Hanisch, S. E., Twomey, C. D., Szeto, A. C., Birner, U. W., Nowak, D., & Sabariego, C. (2016). The effectiveness of interventions targeting the stigma of mental illness at the workplace: a systematic review. *BMC psychiatry*, *16*(1), 1-11.
23. Kaur, A., Kallakuri, S., Kohrt, B. A., Heim, E., Gronholm, P. C., Thornicroft, G., & Maulik, P. K. (2020). Systematic review of interventions to reduce mental health stigma in India. *Asian journal of psychiatry*, 102466.

### Inclusion tool for study screening

**Tool for study selection**

1. Studies must focus on any type of mental health stigma or discrimination (social stigma, self-stigma, anticipated, perceived, experiences, structural stigma and discrimination), or relate to negative attitudes, beliefs or behaviours towards people with mental health issues or mental health in general.
   - - - *Exclude*: studies that only focus on stigma of:
         - Substance misuse
         - Neurological conditions
         - Physical health conditions
         - Other stigmatised social characteristics (sexuality, nationality, gender)
2. Study must be an intervention study. All experimental designs included

- *Exclude*: observational studies

1. Intervention should be indirect (blog posts, photo-voice, theatre performance, website with personal experience stories, imagined). Intervention that was co-produced with people with lived experience should be included too as long as those individuals are not directly talking or engaging with participants face-to-face.

- *Exclude*: interventions that have a person with mental health problem directly speaking to participants.

1. Must be conducted in a LMIC

- *Exclude*: HIC
  - Paper should include stigma-related outcome measure (E.g. Bogardus Social Distance Scale

### Included fields for data extraction

**General information**

| *Date of data extraction* |
| --- |
| *Title* |
| *Authors* |
| *Year of publication* |

**General Study Characteristics**

| *Study design* |
| --- |
| *Country* |
| *Setting (city, village, area)* |
| *Primary aim* |
| *Secondary aims* |
| *Target population* |
| *Target condition (general MH, depression etc)* |
| *Type of stigma targeted (according to Pescosolido et al [6])* |

**Study methods**

| *Timing of the intervention* |
| --- |
| *Mean age of participants* |
| *% female of participants* |
| *Education level* |
| *Sample size (enrolled)* |
| *Sample size (intervention)* |
| *Sample size (control)* |
| *Sample size (2^nd^ comparator arm)* |
| *Sample size (analysed data)* |
| *Other socio-demographic info* |
| *Inclusion criteria* |
| *Exclusion criteria* |
| *Method of recruitment* |
| *Follow up timing* |

**Intervention characteristics**

| *Rationale for indirect contact* |
| --- |
| *Main intervention methods/components (online, video, theatre, storytelling, imagined etc)* |
| *Mix of interventions (indirect social contact only or ISC and other intervention)* |
| *Information about the indirect social contact in the paper* |
| *Intervention development information* |
| *Duration* |
| *Number of sessions/ Frequency* |
| *Delivery agent training/qualification* |
| *Involvement of service users/ people with lived experiences* |
| *Use of technology besides computer/laptop* |
| *Content detail (education, myth busting, motivational, recovery, etc)* |
| *Economic information* |
| *Other relevant intervention notes* |
| *Provided link or materials* |

**Results**

| *Outcome 1: scale/measure* |
| --- |
| *Outcome 1: definition and important detail* |
| *Outcome 1: measure validation?* |
| *Outcome 1: baseline mean (SD)* |
| *Outcome 1: post-intervention mean (SD)* |
| *Outcome 1: follow-up mean (SD)* |
| *Main results found for outcome 1* |
| *Outcome 2: scale/measure* |
| *Outcome 2: definition and important detail* |
| *Outcome 2: measure validation?* |
| *Outcome 2: baseline mean (SD)* |
| *Outcome 2: post-intervention mean (SD)* |
| *Outcome 2: follow-up mean (SD)* |
| *Main results found for outcome 2* |
| *Outcome 3: scale/measure* |
| *Outcome 3: definition and important detail* |
| *Outcome 3: measure validation?* |
| *Outcome 3: baseline mean (SD)* |
| *Outcome 3: post-intervention mean (SD)* |
| *Outcome 3: follow-up mean (SD)* |
| *Main results found for outcome 3* |
| *% of complete outcome data (for all points)* |
| *Overall effectiveness for stigma related outcomes* |
| *Evidence of effectiveness* |
| *Other important notes on results (by AM or citations from text)* |

**Other**

| *Other comments (regarding funding, limitations, etc)* |
| --- |

**Quality assessment (MMAT) summary**

| *% of criteria met* |
| --- |
| *% of complete outcomes data at all points* |
| *Overall quality* |
| *Comments* |
